# MK8722 initiates early-stage autophagy while inhibiting late-stage autophagy via FASN-dependent reprogramming of lipid metabolism

**DOI:** 10.7150/thno.83051

**Published:** 2024-01-01

**Authors:** Luhui Wang, Haiyan Zhu, Zhehao Shi, Bo Chen, Huirong Huang, Ganglian Lin, Jiacheng Li, Haitao Yu, Shihao Xu, Gang Chen, Rongying Ou, Chunxiu Dai

**Affiliations:** 1 Department of Ultrasonography, The First Affiliated Hospital of Wenzhou Medical University, Wenzhou 325015, China.; 2 Department of Obstetrics and Gynecology, The First Affiliated Hospital, Wenzhou Medical University, Wenzhou 325015, China.; 3 Department of Gynecology, Shanghai First Maternity and Infant Hospital, Tongji University School of Medicine, Shanghai 201204, China.; 4 Department of Dermatology, The First Affiliated Hospital of Wenzhou Medical University, Wenzhou 325015, China.; 5 Department of Hepatobiliary Surgery, The First Affiliated Hospital of Wenzhou Medical University, Wenzhou 325015, China.; 6 Department of Traditional Chinese Medicine, The First Affiliated Hospital of Wenzhou Medical University, Wenzhou 325015, China.; 7 Wenzhou Municipal Key Laboratory of Pediatric Pharmacy, Department of Pharmacy, The Second Affiliated Hospital and Yuying Children's Hospital of Wenzhou Medical University, Wenzhou 325027, China.

**Keywords:** epithelial ovarian cancer, FASN, MK8722, mitochondrial fission, STX17-SNAP29-VAMP8

## Abstract

**Background and objective:** Epithelial ovarian cancer (EOC) is associated with latent onset and poor prognosis, with drug resistance being a main concern in improving the prognosis of these patients. The resistance of cancer cells to most chemotherapeutic agents can be related to autophagy mechanisms. This study aimed to assess the therapeutic effect of MK8722, a small-molecule compound that activates AMP-activated protein kinase (AMPK), on EOC cells and to propose a novel strategy for the treatment of EOC.

**Purpose:** To explore the therapeutic effects of MK8722 on EOC cells, and to elucidate the underlying mechanism.

**Methods and results:** It was found that MK8722 effectively inhibited the malignant biological behaviors of EOC cells. *In vitro* experiments showed that MK8722 targeted and decreased the lipid metabolic pathway-related fatty acid synthase (FASN) expression levels, causing the accumulation of lipid droplets. In addition, transmission electron microscopy revealed the presence of autophagosome-affected mitochondria. Western blotting confirmed that MK8722 plays a role in activating autophagy upstream (PI3K/AKT/mTOR) and inhibiting autophagy downstream via FASN-dependent reprogramming of lipid metabolism. Plasmid transient transfection demonstrated that MK8722 suppressed late-stage autophagy by blocking autophagosome-lysosome fusion. Immunofluorescence and gene silencing revealed that this effect was achieved by inhibiting the interaction of FASN with the SNARE complexes STX17-SNP29-VAMP8. Furthermore, the antitumor effect of MK8722 was verified using a subcutaneous xenograft mouse model.

**Conclusion:** The findings suggest that using MK8722 may be a new strategy for treating EOC, as it has the potential to be a new autophagy/mitophagy inhibitor. Its target of action, FASN, is a molecular crosstalk between lipid metabolism and autophagy, and exploration of the underlying mechanism of FASN may provide a new research direction.

## Introduction

Epithelial ovarian cancer (EOC) is the most common type of ovarian cancer (OC), one of the four malignancies of the female genital tract 1. At present, EOC remains one of the deadliest cancer types in women and is the seventh most common cancer in females globally 2. Due to its asymptomatic nature, the EOC is typically identified at an advanced stage and is associated with a poor five-year survival rate 3. Cytoreductive surgery is the most prevalent therapeutic approach for OC 4. However, only a small percentage of ovarian cancer patients are eligible for curative tumor resection, due to many of them suffering from metastatic disease. The extensive use of platinum-based drugs has enhanced the prognosis of OC patients, although their side effects and risk of innate/acquired resistance restrict their efficacy, resulting in low survival rates 5-7. Therefore, it is vital to explore drug resistance mechanisms in order to increase OC patients' survival.

Autophagy is a selective or non-selective dynamic cellular degradation process of mammalian cells 8, 9, during which target molecules or organelles are engulfed into autophagosomes (early-stage autophagy), subsequently, autophagic vesicles are fused with lysosomes, creating autolysosomes where the cargo is eventually degraded by the action of lysosomal hydrolases (late-stage autophagy) 10, 11. Autophagy is engaged in widespread human diseases, including cancer. A growing body of proof has linked alterations in autophagy to the ontogeny of neoplasms and modified reactions to anti-cancer therapies 12, 13, suggesting that a combined application of autophagy modulators with various therapeutic agents could synergistically inhibit tumor growth 14. As a high proportion of tolerance of cancer cells to the majority of chemotherapeutic agents is attributed to autophagy, suppression of autophagy alongside chemotherapy constitutes a novel therapeutic strategy. While chloroquine (CQ) and its derivatives, in addition to other more efficacious autophagy-specific suppressors, have been of scientific interest, they have not been widely applied to clinical practice 15-18. Consequently, it is highly relevant to develop a novel autophagic inhibitor with the potential for clinical implementation.

MK8722 (C_24_H_20_ClN_3_O_4_) is a highly effective and straightforward small-molecule compound activator of 5'-adenosine monophosphate-activated protein kinase (AMPK) in mammals 19-21 that has been exploited due to its broad spectrum of biological activities (Figure 3A). AMPK is part of a metabolite-sensing protein kinase series; studies have demonstrated that AMPK regulates several metabolic pathways and might be a prospective therapeutic target for managing cancer 22-24. AMPK activates autophagy via a dual “fail-safe” mechanism 25-27. However, the curative effects of MK8722 on human EOC and its underlying mechanism have not been fully clarified.

In this study, we researched the promising potential of MK8722 for treating human EOC, and attempted to elucidate its accurate molecular regime. We discovered the roles of MK8722 in restraining malignant biological behavior and fostering cell death in human EOC cells; mechanistically, we observed that MK8722 could activate upstream autophagy (PI3K/AKT/mTOR), downregulate the expression level of fatty acid synthase (FSAN), and repress downstream autophagy by obstructing autophagosome-lysosome fusion. Meanwhile, MK8722 also suppresses lipophagy and affects mitochondrial mitosis, leading to a dramatic accumulation of autophagosomes/lipid droplets/ROS. Importantly, this unexpected role can likely be explained by a hidden mutual interplay between FASN and the SNARE (STX17-SNAP29-VAMP8) compounds, as the diminution of FASN lowered the makeup of the SNARE complexes.

MK8722 activates early autophagy but inhibits late autophagy by inducing FASN-dependent reprogramming of lipid metabolism. The healing action of MK8722 was corroborated by a further demonstration in a nude mouse xenograft model. Our discovery demonstrates the validity of using MK8722 for treating human epithelial ovarian cancer and possibly paves the way for a new therapeutic approach. FASN, its site of action, is a molecular perturbation between lipid metabolism and autophagy, and the underlying mechanism of the freshly developed FASN may represent a new research challenge.

## Materials and Methods

### Reagents and Antibodies

The MK8722 (C_24_H_20_ClN_3_O_4_, purity > 99.37%, #1394371-71-1), Rapamycin (#53123-88-9), Bafilomycin A1 (#88899-55-2) and Nile red (#7385-67-3) were purchased from MCE Co., Ltd. (Beijing, China). The ROS assay kit, cell counting kit-8 (CCK-8), and cytotoxicity assay kit were obtained from Dojindo Laboratories (Kumamoto, Japan). Fetal bovine serum (FBS) was sourced from Gibco (Grand Island, NY, USA). The BCA Protein Assay kit (#P0010), RNAeasyTM Animal RNA Isolation kit with Spin Column, and the hematoxylin and eosin staining kit were purchased from Beyotime Institute of Biotechnology (Shanghai, China). SYBR-Green Master Mix kits and PrimeScriptTM RT Master Mix were acquired from Takara Bio, Inc. (Shiga, Japan). Oil red O solution was obtained from Solarbio Co., Ltd. (Beijing, China). Lipofectamine 3000 transfection reagent (#GK20006) was obtained from Glpbio Technology Co., Ltd. The TG test kit (#A110-2-1) was obtained from Nanjing Jiancheng Bioengineering Institute (Nanjing, China). mtSOX Deep Red (MT14), JC-1 MitoMP Detection Kit (MT09), and Hoechst 33258 (H342) were purchased from Dojindo (Shanghai, China). Furthermore, the following antibodies were commercially available: Ki67 (16667s; Abcam, Cambridge, UK), ATG7 (ab133528; Abcam), p62/SQSTM1 (ab207305; Abcam), BECN1 (ab207612; Abcam), LC3B (NB600-1384 (NOVUS), 3868S (Cell Signaling Technology)), LAMP2 (66301-1-Ig; Proteintech), FASN (3180s; Cell Signaling Technology), mammalian target of rapamycin (mTOR) (2983s; Cell Signaling Technology), PI3K (4249s; Cell Signaling Technology), AKT (9272s; Cell Signaling Technology), p-mTOR (5536s; Cell Signaling Technology), p-PI3K (AF7421; Affinity, Cincinnati, OH, USA), p-AKT (4060s; Cell Signaling Technology), STX17 (ab229646; Abcam; sc-518187; Santa Cruz Biotechnology), endobrevin (G-12) (VAMP8) (sc-166820; Santa Cruz Biotechnology, 15546-1-AP; proteintech), glyceraldehyde 3-phosphate dehydrogenase (GAPDH) (ab8245; Abcam), CDK1 (19532; Proteintech), Cyclin D1 (26939; Proteintech), Cyclin A2 (18202; Proteintech), and β-actin (4070s; Cell Signaling Technology). Secondary antibodies used in the immunofluorescence assay included AF488-anti-mouse (Invitrogen, Carlsbad, CA, USA), AF488-anti-rabbit (Invitrogen), AF594-anti-mouse (Invitrogen), and AF594-anti-rabbit (Invitrogen). Furthermore, anti-rabbit PLUS (#DUO92002, Sigma, St. Louis, MO, USA) and anti-mouse MINUS (#DUO92004, Sigma, St. Louis, MO, USA) probes were utilized. Transwell chambers, cell culture dishes, bottles, six-well plates, 96-well plates, and other cell culture supplies purchased from NEST Biotechnology (Wuxi, China).

### Cell culture and proliferation assay

The human epithelial ovarian cancer (EOC) cell lines (A2780, OV90, SKOV3) were obtained from Shanghai Zhong Qiao Xin Zhou Biotechnology Co., Ltd. (Shanghai, China). A2780 cells were cultured in complete dulbecco's modified Eagle's medium (DMEM) containing 100 µg/ml streptomycin, 100 U/ml penicillin, and 10% fetal bovine serum (FBS) at 37 °C with 5% CO_2_. OV90 cells were cultured in a complete mixture of MCDB 105 and 42.5% M199 basal medium supplemented with 100 µg/ml streptomycin, 100 U/ml penicillin, and 15% FBS at 37 °C with 5% CO_2_. SKOV3 cells were cultured in a complete RPMI-1640 medium containing 100 µg/ml streptomycin, 100 U/ml penicillin, and 15% FBS at 37 °C with 5% CO_2_. For the evaluation of the effects of MK8722 on A2780 and OV90 cells, a CCK-8 kit was used. Cells were seeded at a density of approximately 3×10^3^ cells/well in 96-well plates and incubated for 24 h. Subsequently, the cells were treated with MK8722 for 48 h. The CCK-8 solution was diluted in the culture medium at a ratio of 1:10 and added to the 96-well plates. After 2 h of incubation, cell viability was determined by comparison to non-treated control cells. The half-maximal inhibitory concentration of MK8722 (IC_50_) was calculated. Each experiment was performed in five replicate wells, with three independent experiments conducted for each cell line. To validate the effects of MK8722 on EOC cell lines, real-time cell analysis (RTCA) using label-free impedance monitoring was employed. The xCELLigence RTCA S16 instrument (Agilent Technologies Inc., Santa Clara, CA, USA) was utilized to quantify cell proliferation, morphology changes, and attachment quality in a non-invasive manner. Cells were seeded on E16 plates (E plates; Agilent Technologies Inc.) to monitor real-time cytotoxicity responses. A2780 and OV90 cells were seeded at inoculation densities of 5.0 × 10^3^ cells/well and 8 × 10^3^ cells/well, respectively. Impedance recordings were collected every 15 min 28. After 24 h of inoculation, MK8722 was added to A2780 and OV90 cells separately at concentrations of 0, 5, 10, 20, 40, 60, 80 and 100 μM.

### Colony formation assay

A total of approximately 500 A2780 or OV90 tumor cells were inoculated into each well of a 6-well plate. Subsequently, 0, 20, and 40 μM of MK8722 were added and incubated for 48 h. After one week, the cell clusters were fixed with 4% paraformaldehyde for 20 min. Cell colony formations were assessed by staining with crystal violet at 37 °C for 20 min 29.

### Cell migration assays

According to the protocol of the wound healing assay, exponential growth EOC cells were inoculated into 6-well plates and cultivated at 37 °C until the plates were almost full. In the next step, 200 µL of yellow spearhead was painted onto the culture area to generate a single linear gap through the confluent cell monolayer. The leftover solution was then cleaned up with phosphate-buffered saline (PBS), and the culture medium containing 0, 20, and 40 μM MK8722 was added. After 2 days, images of the cultured area were captured using a microscope.

For the Transwell assay, Transwell chambers with 8 µm pore polycarbonate membrane filters were used. EOC cells (1×10^5^) were placed in the upper chamber of the Transwell by adding 200 µL of serum-free culture medium containing different concentrations of MK8722. The bottom chamber was filled with 600 µL of DMEM containing 20% FBS. After 2 days, the cells that migrated through the pores were fixed with paraformaldehyde for 10 min and stained with crystal violet for 20 min. At least 5 random field images were captured for each chamber (zoom, ×100). All experiments were performed independently with triplicate samples.

### Western blotting

A2780 and OV90 cells were cultured in petri dishes and treated with 0, 20, or 40 μM MK8722 for 48 h, or with 20 μM MK8722 for 6, 12, 24, 36, and 48 h. The treated and non-treated cells were then lysed in cell lysis solution for a minimum of 30 min on ice. The cell lysates were centrifuged at 12,500 ×g at 4 °C for 15 min, and the supernatant was collected. The total protein concentration was determined using the BCA assay kit. The proteins were mixed with 5x loading buffer and PBS, and boiled at 100 °C for 10 min. After denaturation, equal amounts of proteins were separated by SDS-PAGE and transferred to PVDF membranes. It should be noted that proteins of different molecular weights require different transfer times. For example, a large molecular weight protein like FASN requires a fast transfer time of 38 min to ensure clear bands. After blocking the membranes with QuickBlock™ closure buffer for 30 min at room temperature, the membranes were incubated overnight with primary antibodies at 4 °C. After three washes, immunoreactivity was detected using enhanced chemiluminescence reagents by incubating the membranes with appropriate secondary antibodies at room temperature for 1 h. Each experiment was performed in triplicate and repeated in three independent sessions.

### The structure of MK8722 and screening of targets for pharmacodynamics groups

The SDF file containing the 3D structure of MK8722 (Compound CID: 89558344) was obtained from the PubChem database and converted to mol2 format using Open Babel software. Subsequently, the mol2 file was submitted to the PharmMapper server, where all protein target libraries were selected with default parameter values. The PharmMapper server matched MK8722 with its pharmacophore model in the database and exported the results, which were then further annotated with protein and coding genes using UniProt. Duplicate data were filtered and backed up, and the screening results were analyzed using FitScore software.

### Kyoto Encyclopedia of Genes and Genomes (KEGG) pathway enrichment analysis

To gain further insights into the functions of the identified pharmacophore targets, Kyoto Encyclopedia of Genes and Genomes (https://www.genome.jp/kegg/) pathway analyses were conducted. The clusterProfiler package in R was used for the analysis, and pathways with a *p*-value < 0.05 were considered significantly enriched pathways.

### Molecular docking verification of MK8722 with target genes

The 2D molecular structure of MK8722 was converted into a 3D structure using Chem3D software. The target genes were processed using AutoDockTool to perform desolvation, hydrogenation, and charge addition. Subsequently, molecular docking of MK8722 with the target genes was performed using PyMOL mapping.

### Oil red O staining

A2780 and OV90 cells cultured on round-shaped coverslips were seeded into 12-well plates, while tissue samples were embedded in OCT compound and sectioned into 10 µm sections. Cells or tissue sections were washed with PBS, fixed with 4% paraformaldehyde for 20 min, and then washed twice with distilled water. Subsequently, they were immersed in 60% isopropanol for 5 min. Cells were stained with oil red O solution (stock solution/ddH2O, 3:2) for 5 min. Finally, cell nuclei were carried out with hematoxylin for 3 min and followed by bluing returned to blue for 3 s, and sealed with glycerol. Images were captured using a fluorescence microscope.

### Measurement of triglyceride (TG) content

The TG content was quantified using the TG assay kit. Cells were processed and handled according to the manufacturer's instructions. The absorbance of cells was measured using an enzyme marker, and data analysis was performed using GraphPad Prism software.

### Transmission electron microscopy (TEM)

Cells were cultured as indicated and fixed with 2.5% glutaraldehyde at 4 °C overnight. They were then post-treated with 2% osmium tetroxide at room temperature for 1.5 h. Following fixation, the cells were embedded in paraffin and stained with uranyl acetate/lead citrate. Standard procedures were followed for transmission electron microscopy (Wenzhou Medical University, Wenzhou, China).

### Nile red staining

A2780 and OV90 cells were transfected with EGFP-LC3. After 48 h of treatment with or without MK8722, cells were washed thrice with PBS and incubated with the Nile red probe according to the manufacturer's instructions. After washing with PBS, cells were observed and photographed using laser confocal scanning microscopy (CLSM).

### Determination of reactive oxygen species (ROS) levels and flow cytometry

Cellular ROS levels were assessed using a ROS assay kit. Treated and untreated A2780 and OV90 cells were vaccinated into 6-well plates (24 mm × 24 mm), allowing them to reach 60-70% confluency. After 48 h of treatment, cells were washed thrice with PBS and incubated with the DCFH-DA probe following the manufacturer's instructions. After washing with PBS, cells were observed and photographed under an inverted fluorescence microscope. For flow cytometry analysis, cells were processed, acquired, and stained with DCFH-DA according to the manufacturer's instructions. The cells were then analyzed using a flow cytometer (Beckman Coulter Inc., Brea, CA, USA)**.**

### Measurement of the mitochondrial membrane potential

The mitochondrial membrane potentials were determined using the JC-1 MitoMP Detection Kit. Treated and untreated A2780 and OV90 cells were vaccinated onto confocal dishes and allowed to reach 60-70% confluency. After 48 h of treatment, cells were washed thrice with PBS and incubated with JC-1 Dye according to the manufacturer's instructions. After washing with PBS, cells were observed and photographed using CLSM. For flow cytometry analysis, cells were processed, acquired, and stained with JC-1 according to the manufacturer's instructions. The cells were then analyzed using a flow cytometer (Beckman Coulter Inc., Brea, CA, USA).

### Intracellular ROS measurement

The levels of mitochondrial superoxide were determined using the mtSOX Deep Red assay kit. Treated and untreated A2780 and OV90 cells were seeded onto confocal dishes and allowed to reach 60-70% confluency. After 48 h of treatment, cells were washed thrice with PBS and incubated with the mtSOX Deep Red probe according to the manufacturer's instructions. After washing with PBS, cells were observed and photographed using CLSM. For flow cytometry analysis, cells were processed, acquired, and stained with mtSOX according to the manufacturer's instructions. The cells were then analyzed using a flow cytometer (Beckman Coulter Inc., Brea, CA, USA).

### Cell cycle analysis

For cell cycle analysis, A2780 and OV90 cells were seeded in Petri dishes and treated with different concentrations of MK8722 for 48 h. Cells were washed three times with PBS, digested into cell suspensions, and then washed three times with PBS again. They were then mixed with pre-cooled 75% ethanol and stored at 4 °C for 4 h. After washing with PBS three times, 7-AAD reagent (BD Pharmingen, SD, USA) was added to the cells, and the suspension was incubated for 20 min. The DNA content of each cell cycle phase was measured. To detect apoptosis in A2780 and OV90 cells, cell suspensions obtained using the same method as mentioned above were incubated with the FITC Annexin V apoptosis detection kit (BD Pharmingen, SD, USA) for 20 min. Subsequently, the apoptosis ratio of each group was determined.

### Transfection and RNA interference

Transfection was performed using Lipofectamine 3000 transfection reagent following the manufacturer's protocol. The plasmids EGFP-LC3 and RFP-MITO were constructed by GeneChem Co. (Shanghai, China). After 24-48 h of incubation, the transfection solution was replaced with a fresh complete culture medium. For RNA interference, the FASN-siRNA plasmid was constructed by Suzhou GenePharma Co., Ltd. (Suzhou, China), and FASN-siRNA was introduced into A2780 and OV90 cells according to the manufacturer's specifications.

### Immunofluorescence

A2780 and OV90 cells were seeded on round-shaped coverslips in 12-well plates and stained for lysosomes using LysoTracker Red solution (#C1046; Molecular Probes Inc., Eugene, OR, USA) under different experimental conditions. Cells were fixed with 4% paraformaldehyde for 10 min, washed with PBS, blocked with bovine serum albumin (BSA), and then incubated overnight at 4 °C with primary antibodies (Ki67 (1:200), STX17 (1:50), and VAMP8 (1:500)) separately. Afterward, appropriate secondary antibodies were applied for 1 h. Cell nuclei were stained with 4′,6-diamidino-2-phenylindole (DAPI) solution for 5 min. Finally, the cells were observed using an ortho-fluorescence microscope or a laser scanning confocal microscope (Leica, Wetzlar, Germany). The area of Ki67-positive cells and the localization of each STX17 and VAMP8 cluster were quantified using ImageJ software (National Institute of Health, Bethesda, MD, USA).

### RNA extraction and quantitative polymerase chain reaction (qPCR) assay

Total RNA was extracted from A2780 and OV90 cells treated with 0, 20, and 40 μM MK8722 for 48 h using the RNAeasyTM Animal RNA Isolation kit following the manufacturer's instructions. The total RNA was then reverse transcribed into cDNA using the PrimeScriptTM RT Master Mix software. qPCR was performed on a PCR detection system (ABI Prism 7500 system; Applied Biosystems, Waltham, MA, USA) under standard conditions using the SYBR-Green Master Mix kit. All reactions were repeated three times, and the results were normalized to β-actin expression. Relative gene expression was calculated using the 2^-ΔΔCt^ method. The primers used for qPCR were as follows: 5'-GTGGTGGGCTTGGTGAACTGTC-3' (FASN F), 5'-AGGTGCTGCTGAGGTTGGAGAG-3' (FASN R), 5'-TGATTGAGTCCCTCTCCCAGATGC-3' (P62 F), 5'-CCGCTCCGATGTCATAGTTCTTGG-3' (P62 R), 5'-GTCAGCGTCTCCACACCAATCTC-3' (LC3B F), 5'-ACAATTTCATCCCGAACGTCTCCTG-3' (LC3B R), 5'-CCTGGCACCCAGCACAAT-3' (β-actin F), 5'-GGGCCGGACTCGTCATAC -3' (β-actin R).

### Co-immunoprecipitation (Co-ip)

A2780 and OV90 cells were treated with or without Rapa (1 μM) in the presence of normal or silenced FASN for 6 h. After washing the cells with cold PBS, they were lysed with cell lysis buffer containing a protease inhibitor. The cells were thoroughly disrupted through methods such as cell scraping and then centrifuged to remove cell debris and nucleic acids. The cell lysate was incubated with specific antibodies at 4 °C to allow complex formation. Protein A/G agarose beads pre-bound to antibodies were used for precipitation. The immunoprecipitation complexes were washed with PBS to remove non-specifically bound proteins. and then separated by SDS-PAGE electrophoresis and transferred onto a membrane through protein blotting. Finally, specific antibody probes were used for Western blot analysis of the target protein.

### Proximity ligation assay (PLA) assay

The Duolink® PLA assay was performed according to the manual. Briefly, A2780 and OV90 cells were treated as indicated and stained with mouse anti-VAMP8 and rabbit anti-STX17 LDHA antibodies as described for the immunofluorescent staining. Duolink® PLA was then conducted using the anti-rabbit PLUS (#DUO92002, Sigma, St. Louis, MO, USA) and anti-mouse MINUS (#DUO92004, Sigma, St. Louis, MO, USA) probes. Following probe incubation, ligation, and amplification, the cells were observed and imaged using a confocal microscope.

### Establishment of a xenograft mouse model

Female BALB/c nude mice (6-8 weeks old) were obtained from the First Affiliated Hospital of Wenzhou Medical University and housed in specific pathogen-free (SPF) facilities with feed provided by the Experimental Animal Center of the First Affiliated Hospital of Wenzhou Medical University. A total of 1×10^6^ A2780 or OV90 cells were subcutaneously injected into the right midline of the nude mice. Starting from day 6, tumor growth was monitored every 3 days, with measurements taken for body weight, tumor weight, and tumor volume. On day 12, when the tumor growth reached approximately 120 mm^3^, nude mice bearing tumors were randomly divided into 4 groups (5 mice per group). The control solvent and MK8722 (30 mg/kg) were administered via gavage every 2 days to the mice in the control and experimental groups. Tumor width and length in the nude mice were measured every 2 days, and tumor volume was calculated using the formula V = (length×width^2^)/2. Animal experiments were approved by the First Affiliated Hospital of Wenzhou Medical University and adhered to the National Institutes of Health Guide for the Use of Laboratory Animals (NIH 1985:86-23).

### Immunohistochemistry (IHC)

The expression levels of Ki-67 and LC3B proteins in subcutaneous tumors of nude mice were evaluated using IHC. Paraffin-embedded tissues were sectioned into 5 μm slices. The tissue slices were dried in a desiccation chamber at 70 °C for 2 h, dewaxed with xylene, and rehydrated with graded ethanol. The slides were then placed in 10 mM citrate buffer (pH 6.0) and heated in a microwave oven at high temperature for 5 min. This antigen retrieval process was repeated for 2 to 3 cycles. Endogenous peroxidase was blocked with hydrogen peroxide, and cells were incubated with 5% BSA for 1 hour to reduce nonspecific staining. Subsequently, the slices were incubated overnight at 4 °C with primary antibodies against Ki-67 or LC3B. Immunostaining was performed with a secondary antibody labeled with horseradish peroxidase at 37 °C for 1 h. After applying DAB chromogen, staining was carried out with hematoxylin for 3 min, followed by a 3-second blue counterstain. Images were captured using a histological microscope, and the percentage of positive area was calculated using ImageJ software.

### Acute toxicity studies

Vein blood was collected from two groups: the control group and the experimental group. The blood was centrifuged at 3000-5000 rpm for 10-15 min at 4 °C, and the supernatant was venous serum. Subsequently, 200 µL of venous serum from nude mice was transferred to the CATALYST CHEM 10 kit (CAS: 98-11005-01) and inserted into the CATALYST ONE automated animal biochemical analyzer following the manufacturer's instructions to detect the levels of biochemical parameters in the serum, as previously described 28.

### Statistical analysis

Data analysis was performed using GraphPad Prism 8.0 software (GraphPad Software Inc., San Diego, CA, USA) and the R 4.1.3 programming language. Data were presented as mean ± standard deviation (SD), while categorical variables were presented as frequency (percentage). Each experiment was repeated three times, with three data points obtained from three independent parallel experiments. For each of the three parallel experiments, at least five distinct images were counted for each group. We selected thirty or more fluorescence images with a cell count exceeding thirty for quantification of fluorescence spots, and the results were calculated in a single-blind manner. Differences in continuous variables between the two groups were evaluated using the t-test or Wilcoxon rank-sum test. One-way analysis of variance (ANOVA) was used to determine differences between the two groups. Categorical variables were examined using the Chi-square test or Fisher's exact test. Pearson correlation analysis was performed to assess the correlation between the expression levels of two genes. A *p*-value of less than 0.05 was considered statistically significant.

## Results

### MK8722 inhibits the growth and migration of EOC cells *in vitro*

**Figure 1:**
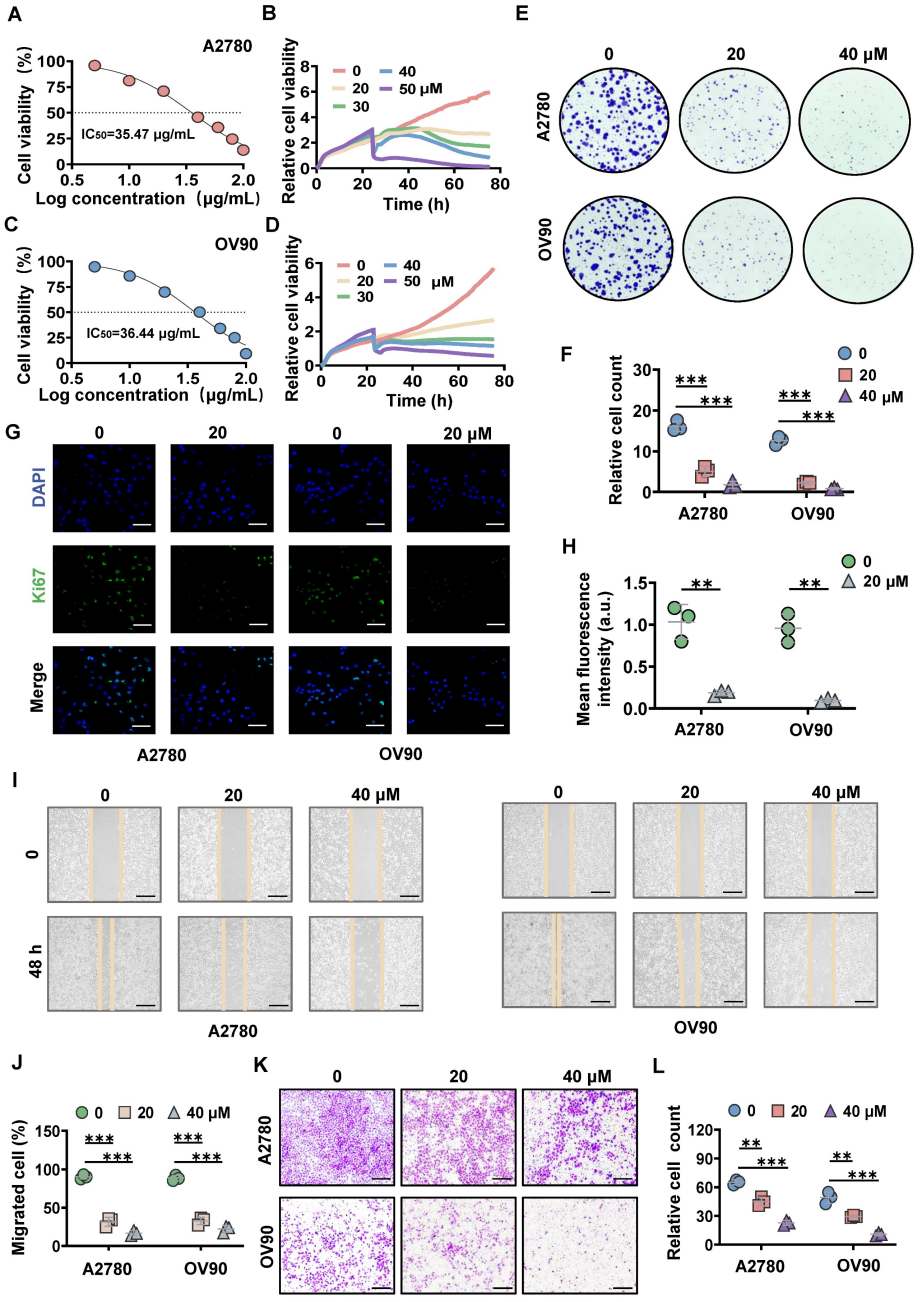
** MK8722 inhibits the growth and migration of EOC cells *in vitro.*** (A, C) A2780 and OV90 cells were incubated with 5, 10, 20, 40, 60, 80 and 100 μM MK8722 (MK) or an equal volume of DMEM for 48 h, respectively. (B, D) A2780 and OV90 cells were exposed to MK, and real-time cell activity was measured using RTCA. (E, F) A2780 and OV90 cells were incubated with MK (20 μM, 40 μM) or an equal volume of DMEM culture solution for 48 h, and a colony formation assay was conducted. (G, H) A2780 and OV90 cells were cultured with the presence of MK (20 μM) or an equal volume DMEM culture for 48 h and then subjected to Ki-67 immunofluorescence analysis. Scale bar: 15 μm. (I, J) A2780 and OV90 cells were plated with 20 μM, 40 μM MK or DMEM for 48 h to observe wound healing. Scale bar: 100 μm. (K, L) Transwell migration assay was used to evaluate the effect of 20 μM and 40 μM MK on the migration of A2780 and OV90 cells. Scale bar: 100 μm. Results were exhibited as mean ± SD; * *p* < 0.05, ** *p* < 0.01, *** *p* < 0.001. The results represent the mean value from three independent experiments (n = 3), and representative pictures are shown.

To estimate the tumor suppressive effect of MK8722, different concentrations of MK8722 were co-incubated with human ovarian cancer cell lines (A2780, OV90, SKOV3). The proliferation ability of the tumor cells was assessed using the CCK-8 assay kit, which revealed that MK8722 inhibited cell growth (Figure 1A, C; Figure S1A). The half-maximal inhibitory concentration (IC_50_) for A2780, OV90, and SKOV3 cells were 35.47, 36.44, and 47.89 μg/mL, respectively. Furthermore, the time analysis showed the reduced survivability of EOC cells when treated with MK8722 in a concentration-dependent manner (Figure 1B, D; Figure S1B). Additionally, the colony formation assay demonstrated that MK8722 suppressed the number of clones formed by A2780, OV90 and SKOV3 cells (Figure 1E, F; Figure S1C). Moreover, immunofluorescence staining for Ki-67, a marker of cell proliferation, revealed a significantly lower number and intensity of fluorescence in the MK8722-treated EOC cells (20 μM) compared to untreated cells (Figure 1G, H; Figure S1D). These results indicated that MK8722 had a dose-dependent inhibitory effect on the proliferation of A2780, OV90 and SKOV3 cells. The migratory ability of EOC cells was assessed using wound healing and Transwell assays. The wound healing assay demonstrated that treatment with 20 and 40 μM of MK8722 slowed down the migration of A2780, OV90 and SKOV3 cells, as evidenced by the intermediate healing area (Figure 1I, J; Figure S1E). The Transwell assay further confirmed these results (Figure 1K, L; Figure S1F). In conclusion, MK8722 exhibited a dose-dependent inhibitory effect on the proliferation and metastasis of EOC cells. Considering the stronger inhibitory effects of MK8722 on A2780 and OV90 than SKOV3, A2780 and OV90 were selected for subsequent experiments.

### MK8722 induces FASN-dependent reprogramming of lipid metabolism

**Figure 2:**
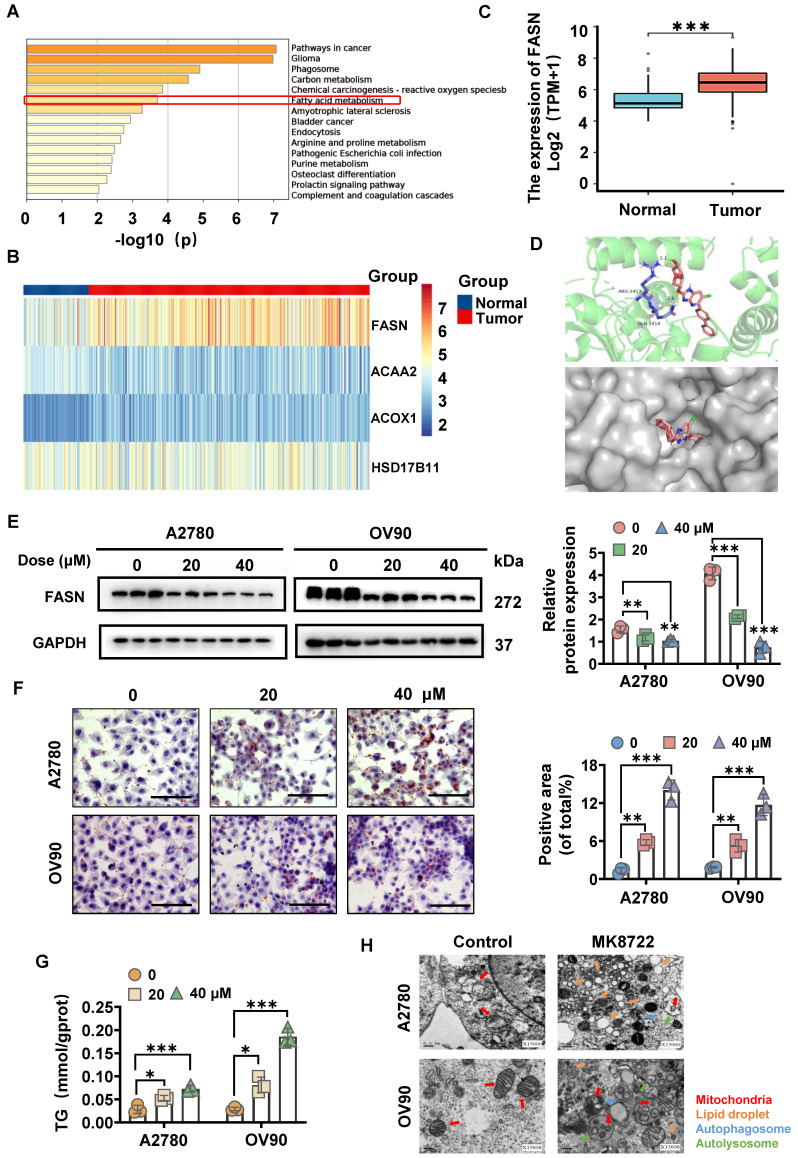
** MK8722 induces FASN-dependent reprogramming of lipid metabolism.** (A) KEGG enrichment analysis revealed pathways related to MK pharmacophore targets. (B, C) Analysis of the expression of lipid metabolism-related drug targets using RNA-seq data downloaded from TCGA and GETx databases. (D) Schematic diagram showing the results of molecular docking between MK and FASN. (E) Western blot analysis of FASN protein expression in A2780 and OV90 cells treated with MK (20 μM, 40 μM) or DMEM for 48 h. The results of WB experiments were quantified and analyzed. (F, G) Oil red O staining and TG content assay experiments were carried out to evaluate the effects of 20 μM and 40 μM MK on lipid droplet formation and triglyceride content in A2780 and OV90 cells. Scale bar: 100 μm. (H) Representative ultrastructure of A2780 and OV90 cells treated with 20 μM MK for 48 h. Red arrows indicate mitochondria; Orange arrows indicate lipid droplets; Blue arrows indicate autophagic vacuoles; Green arrows indicate autophagic lysosomes. Scale bar: 0.5 μm. Results are presented as mean ± SD; * *p* < 0.05, ** *p* < 0.01, *** *p* < 0.001. The results represent the mean value from three independent experiments (n = 3), and representative pictures are shown.

The 3D structure of MK8722 (Compound CID: 89558344) was generated from the PubChem website (https://pubchem.ncbi.nlm.nih.gov/) and evaluated using the PharmMapper server (http://www.lilab-ecust.cn/pharmmapper/), along with the first 300 pharmacophore targets. The pharmacophore targets were annotated and then subjected to the KEGG enrichment analysis, which included glioma, carbon metabolism, phagosome, reactive oxygen species, fatty acid (FA) metabolism, etc., with *p* < 0.05 indicating statistical significance. Among the lipid metabolism pathways, the FA metabolism pathway was selected (Figure 2A). Then, the FA metabolism-related genes were identified utilizing the RNA-sequence of OC tissues (n = 379) and normal ovarian tissues (n = 88) from the Cancer Genome Atlas (TCGA) and GETx databases. As a result, four target genes were confirmed to be differentially expressed between the two groups, with FASN showing statistical significance, suggesting its involvement in OC development (Figure 2B, C). This led us to hypothesize that MK8722 might induce FASN-dependent reprogramming of lipid metabolism. To further investigate this hypothesis, the 3D protein structure (1XKT) of FASN was obtained from the RCSB PDB database (https://www.rcsb.org/), molecular docking was performed using AutoDock Vina, and the results were visualized using Pymol software (Figure 2D). The molecular docking results suggested that FASN could be a target of MK8722 in OC. To confirm this finding, cells were conditioned with various concentrations of MK8722 for 48 h. The results showed that MK8722 significantly downregulated FASN expression in a concentration-dependent manner (Figure 2E).

As shown in Figures 2F and 2G, MK8722 treatment led to a dose-dependent increase in the oil Red O staining area, indicating an increase in lipid droplet content and triglyceride (TG) content. However, this contradicted the results observed in Figure 2E, where a decrease in FASN expression should not lead to lipid droplet accumulation. This discrepancy motivated us to explore the intrinsic mechanism behind lipid droplet degradation. Lipid droplets are dynamic organelles that are synthesized and degraded based on extracellular nutrient conditions. They consist of a neutral lipid core surrounded by a single layer of phospholipids and serve as essential units for lipid storage in cells. After the de novo synthesis of TG and cholesteryl esters (CE) between lipid bilayers in the endoplasmic reticulum, resulting in sufficient lipid accumulation, they are packaged and may be released into the cytoplasm, forming lipid droplets 30, 31. Lipid droplets can be subsequently fragmented through two pathways: lipolysis and lipophagy 32. To investigate the assembly and degradation of lipid droplets in MK8722-treated EOC cells, the ultrastructural changes of A2780 and OV90 cells were observed using transmission electron microscopy (TEM) with findings indicating an increase in lipid droplets in MK8722-treated cells compared to the control group (Figure 2H). Importantly, autophagic vesicles and a small fraction of autophagic lysosomes were observed, suggesting that lipid degradation may occur through autophagy, specifically lipophagy. The uncharacteristic accumulation of lipid droplets might reflect the hindrance of an abnormal course of autophagic flow. Moreover, partially dissected mitochondria (Figure 2H) and few mitotic structures were detected, indicating that these abnormal mitochondria were associated with autophagosomes or surrounded by bilamellar vesicles.

### MK8722 enhances the stability and formation of LC3B-II spots in EOC cells and affects mitosis

**Figure 3:**
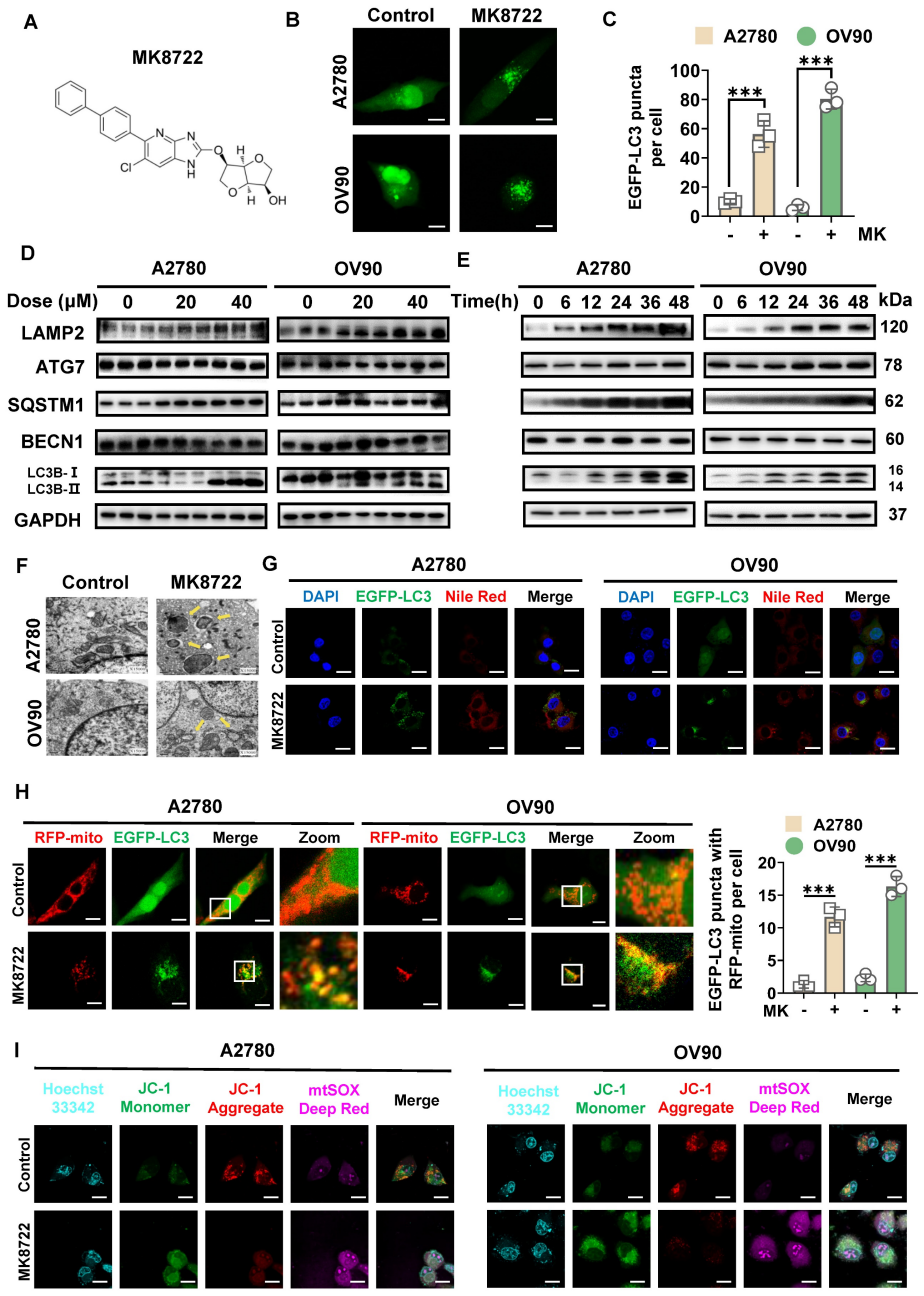
** MK8722 enhances LC3B-II stability and spot formation in EOC cells and affects mitosis.** (A) Chemical structure of MK8722. (B) A2780 and OV90 cells expressing EGFP-LC3 were co-treated with or without MK (20 μM) for 48 h, and EGFP-LC3 spots were observed using confocal microscopy. Scale bar: 10 µm. (C) Quantification of the average number of EGFP puncta per cell in (B) from three independent experiments. (D and E) Cells were exposed to different concentrations of MK for 48 h, or treated with 20 µM MK for various time intervals as indicated. The expression of autophagy-related proteins (LC3B-II/LC3B-I, SQSTM1, BECN1, ATG7, and LAMP2) was assessed by western blot analysis. GAPDH was used as a loading control. (F) Representative transmission electron microscopy images showing the ultrastructure of A2780 and OV90 cells treated with or without MK (20 µM) for 48 h. Yellow arrows indicate autophagic vesicles. Scale bar: 0.5 µm. (G) Confocal microscopy images of A2780 and OV90 cells expressing EGFP-LC3 treated with or without MK (20 µM) for 48 h after staining with Nile Red. Scale bar: 10 µm. (H) Confocal microscopy images of A2780 and OV90 cells treated with or without MK (20 µM) for 48 h after co-expression of RFP-mito and EGFP-LC3. Scale bar: 10 µm. (I) Confocal microscopy images of A2780 and OV90 cells treated with or without MK (20 µM) for 48 h after staining with JC-1 and mtSOX Deep Red. Scale bar: 10 µm. Results are presented as mean ± SD; *** *p* < 0.001. The results represent the mean values from three independent experiments (n = 3), and representative pictures are shown.

To determine the potential impact of MK8722 (Figure 3A) on autophagy in EOC cells, A2780 and OV90 cells transiently expressing EGFP-LC3 were examined using CLSM to evaluate the aggregation of autophagic vesicles (Figure 3B). The MK8722-treated group showed a significant increase in the formation of EGFP-LC3 spots in both A2780 and OV90 cells (Figure 3C). During the dynamic homeostasis of autophagy, LC3/Atg8 was clipped by Atg4 to generate cytoplasmic LC3-I which in turn underwent a pantothenic-like reaction that was associated with PtdEth (PE) to generate the lipidated form of phagocytosis-associated LC3-II, a structural protein of the autophagosome 33. LC3B is a marker of autophagy that is widely utilized to appraise activity 34-36, hence thereafter, the influences of MK8722 on the alteration of LC3B in A2780 and OV90 cells were explored. Western blot analysis revealed that the aggregation of LC3B-II in two EOC cell lines was not only dose-dependent (Figure 3D), but also to a certain extent time-dependent (Figure 3E) under MK8722 treatment. In addition to the LC3B protein, the expression levels of other autophagy-associated proteins, that is, lysosome-associated membrane protein 2 (LAMP2), ubiquitin-binding protein p62/SQSTM1, and pivotal autophagy-promoting proteins (BECN1 and ATG7) were tested by immunoblotting (Figure 3D, E). The results indicated that MK8722 treatment significantly increased the expression levels of LAMP2 and p62/SQSTM1 in a dose- and time-dependent manner. In contrast, the MK8722 treatment did not affect the protein levels of BECN1 or ATG7 (Figure S2A-E). As p62/SQSTM1 plays a role in selective autophagy by linking ubiquitinated proteins to autophagosomes, its cellular levels are negatively correlated with autophagic activity when autophagic flux is suppressed. At the transcriptional level, the expression levels of p62/SQSTM1 and LC3B-II were verified by qRT-PCR, which showed consistency with the protein levels (Figure S2F). Consequently, it can be speculated that MK8722 is an effective inhibitor of autophagic flux.

To further characterize autophagy in MK8722-treated A2780 and OV90 cells, the effect of MK8722 on the accumulation of LAMP2, LC3B-II, and p62/SQSTM1 in the presence or absence of bafilomycin A1 was assessed using western blot analysis. As shown in Figure S2G, MK8722 treatment resulted in the aggregation of LC3B-II and p62/SQSTM1, comparable to that induced by bafilomycin A1. MK8722 treatment also led to a significant increase in LAMP2, in contrast to the effect of bafilomycin A1. However, the combination of MK8722 and bafilomycin A1 did not markedly alter the accumulation of LC3B-II and p62/SQSTM1 compared to MK8722 or bafilomycin A1 alone (Figure S2H). This suggests that MK8722 mediates the accumulation of LC3B-II and SQSTM1 by blocking their degradation through the autophagic lysosomal blockade. Subcellular structural changes were further assessed using TEM, with findings revealing the absence of certain autophagic vesicles in both cell lines after MK8722 treatment, accompanied by minimal or no presence of autophagic lysosomes (Figure 3F).

Based on the observation of accumulated lipid droplets within autophagic vesicles, we previously hypothesized that lipophagy was inhibited. To investigate this further, A2780 and OV90 cells transiently expressing EGFP-LC3 were incubated with a Nile Red fluorescent probe in the presence or absence of MK8722. CLSM analysis showed that the significantly enhanced red fluorescent signal induced by the drug did not overlap with the aggregated green spots, confirming our suspicion (Figure 3G; Figure S2I). We serendipitously discovered the presence of mitochondria within autophagic vesicles, prompting further investigation to elucidate their relationship. To assess the coexistence of autophagic vesicles with mitochondria, we labeled mitochondria with RFP-mito and autophagic vesicles with EGFP-LC3 in A2780 and OV90 cells. The results showed a more pronounced formation of yellow patches indicative of coexistence in MK8722-treated cells, suggesting that MK8722 influenced mitophagy in A2780 and OV90 cells (Figure 3H).

Mitochondria-derived reactive oxygen species (ROS) can act as an upstream signaling molecule for the autophagic process. Conversely, the activation of autophagy can remove mitochondria leaking ROS, thereby downregulating intracellular ROS levels 37. ROS levels were thus assessed to reflect both mitochondrial status and the integrity of the entire autophagic process. Collectively, the pathway enrichment analysis (Figure 2A) also involved ROS and was highly correlated. A DCF fluorescent probe was first utilized to measure ROS levels in MK8722-treated cells versus untreated A2780 and OV90 cells. An apparent increase in ROS fluorescence intensity was observed in both cell lines after MK8722 treatment (Figure S3B), which was consistent with the data obtained from flow cytometry using a DCF probe (Figure S3A), indicating its significant impact on intracellular ROS accumulation (Figure S3C, D). Reactive oxygen species in cells are mainly produced by mitochondria. Therefore, we employed JC-1 and mtSOX co-labeling to evaluate mitochondrial inner membrane potential and oxidative stress status. After drug treatment, we observed a significant decrease in mitochondrial membrane potential and a noteworthy increase in superoxide fluorescence intensity in both cell lines (Figure 3I; Figure S3E, F). Additionally, as there might be overlapping excitation and emission ranges of the fluorophores, we conducted further analysis using flow cytometry, and what we obtained were consistent with the previous findings (Figure S3G-J). These results indicate impaired mitochondrial function and suppressed mitophagy, as he proper execution of mitophagy is crucial for maintaining the mitotic process. To validate these findings, we then assessed the impact of different concentrations of MK8722 on the cell cycle using PI staining and flow cytometry (Figure S3K, L). The results revealed that both cell lines were arrested at the G1 phase under the influence of MK8722, and this arrest exhibited a concentration-dependent pattern. Moreover, there was a significant reduction in the protein expression levels of cell cycle-related markers Cyclin A2, CDK1, and Cyclin D1 in the treated group (Figure S3M, N), indicating an impeded mitotic process and further confirming autophagy inhibition.

### MK8722 suppresses autophagic vesicle-lysosome fusion and inhibits SNARE complexes formation

**Figure 4:**
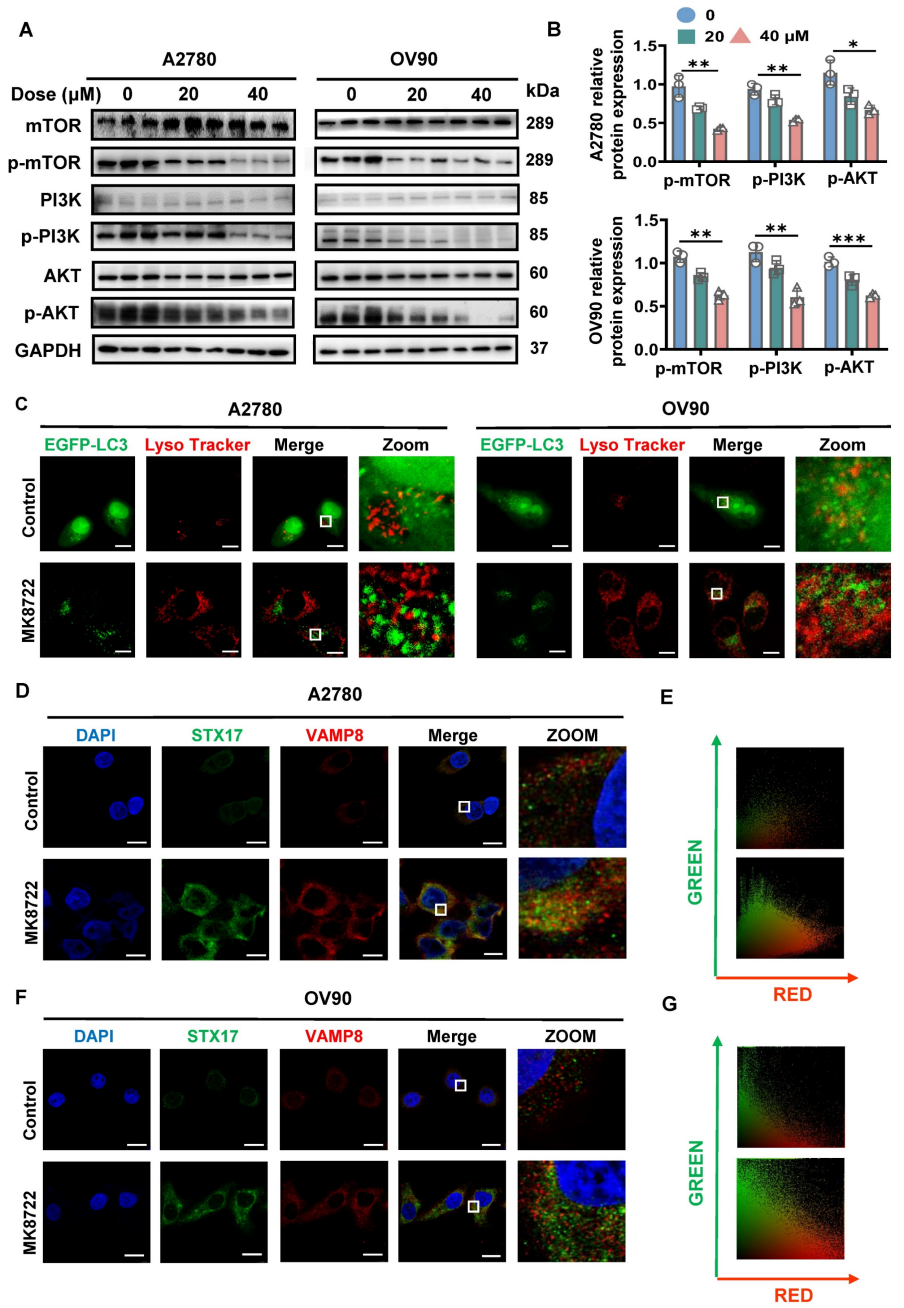
** MK8722 disrupts autophagic vesicle-lysosome fusion and inhibits SNARE complexes formation.** (A, B) A2780 and OV90 cells were cultured in MK (20 μM, 40 μM) or an equal volume of DMEM medium for 48 h, and the expression of autophagy upstream-related proteins (PI3K, AKT, mTOR, p-PI3K, p-AKT, and p-mTOR) was analyzed by western blotting. GAPDH was used as a loading control. Quantification and analysis of western blotting results (p-mTOR, p-PI3K, and p-AKT) were performed. (C) After transient transduction of EGFP-LC3, A2780 and OV90 cells were treated with MK (20 μM) for 48 h. Fluorescence signals were examined under confocal microscopy after staining with LysoTracker Red. Scale bar: 10 μm. (D, F) Representative images showing STX17 (green) and VAMP8 (red) of A2780 and OV90 cells after 48 h of treatment with MK (20 μM). (E, G) Quantitative analysis of the relationship between STX17 and VAMP8 in (C) and (D). Scale bar: 10 μm. Scatter plots were generated using Image Pro Plus software. Results are presented as mean ± SD; * *p* < 0.05, ** *p* < 0.01, *** *p* < 0.001. The results represent the mean values from three independent experiments (n = 3), and representative pictures are shown.

Our previous observations showed that MK8722 treatment increased the levels of p62/SQSTM1 and LAMP2 in various EOC cells, indicating that MK8722 may impede autophagic degradation and act as a potent inhibitor of autophagic flux. During the late stage of autophagy, lysosomes and autophagic vesicles merge to form autophagolysosomes, facilitating degradation. Once this process is blocked, degradation breaks down. To gain further insight into how MK8722 affects the production of autophagic lysosomes by stemming late-stage autophagic flow, western blotting was conducted to measure the expression levels of upstream autophagy-related proteins (PI3K, AKT, and mTOR). The results indicate that p-PI3K, p-AKT, and p-mTOR expression levels were dose-dependently lower in the MK8722-treated group than those in the untreated group (Figure 4A, B), while PI3K, AKT, and mTOR expression levels rarely were changed (Figure S4A, B). This suggests that the upstream PI3K/AKT/mTOR pathway remains unobstructed.

The co-localization of EGFP-LC3 and LysoTracker Red, a fluorescent probe used for specific labeling of lysosomes in living cells, was subsequently examined. It was observed that in MK8722-treated cells, the abundance of EGFP-LC3 puncta increased significantly, while the co-localization with LysoTracker Red was absent (Figure 4C; Figure S4C, D). This indicates that MK8722 suppresses autophagy by inhibiting the fusion of lysosomes with autophagosomes. Since LAMP2 plays a critical role in autophagic vesicle-lysosome fusion, we investigated the effect of MK8722 on LAMP2 expression levels using western blot analysis. Treatment with MK8722 led to a time-dependent increase in LAMP2 protein levels (Figure 3E), suggesting that the mechanism by which MK8722 blocks fusion is not related to a reduction in LAMP2 expression. Autophagosome-lysosome fusion is regulated by multiple factors with the STX17-SNAP29-VAMP8 complex being essential for autophagic vesicle-lysosome fusion, with STX17 being in the autophagosome membrane and VAMP8 in the endosome/lysosome 38-40.

Therefore, the co-localization of STX17 and VAMP8 in A2780 and OV90 cells after treatment with MK8722 or without treatment was examined by immunofluorescence (Figure 4D, F). No coexistence of STX17 and VAMP8 was observed under CLSM. In MK8722-treated cells, both STX17 and VAMP8 displayed stronger fluorescence signals with increased abundance, but they remained unbound (Figure 4E, G). This further suggests that MK8722 disrupts autophagosome-lysosome fusion by preventing the formation of the STX17-SNAP29-VAMP8 complex.

### Silencing of FASN suppresses Rapa-induced SNARE complexes formation

**Figure 5:**
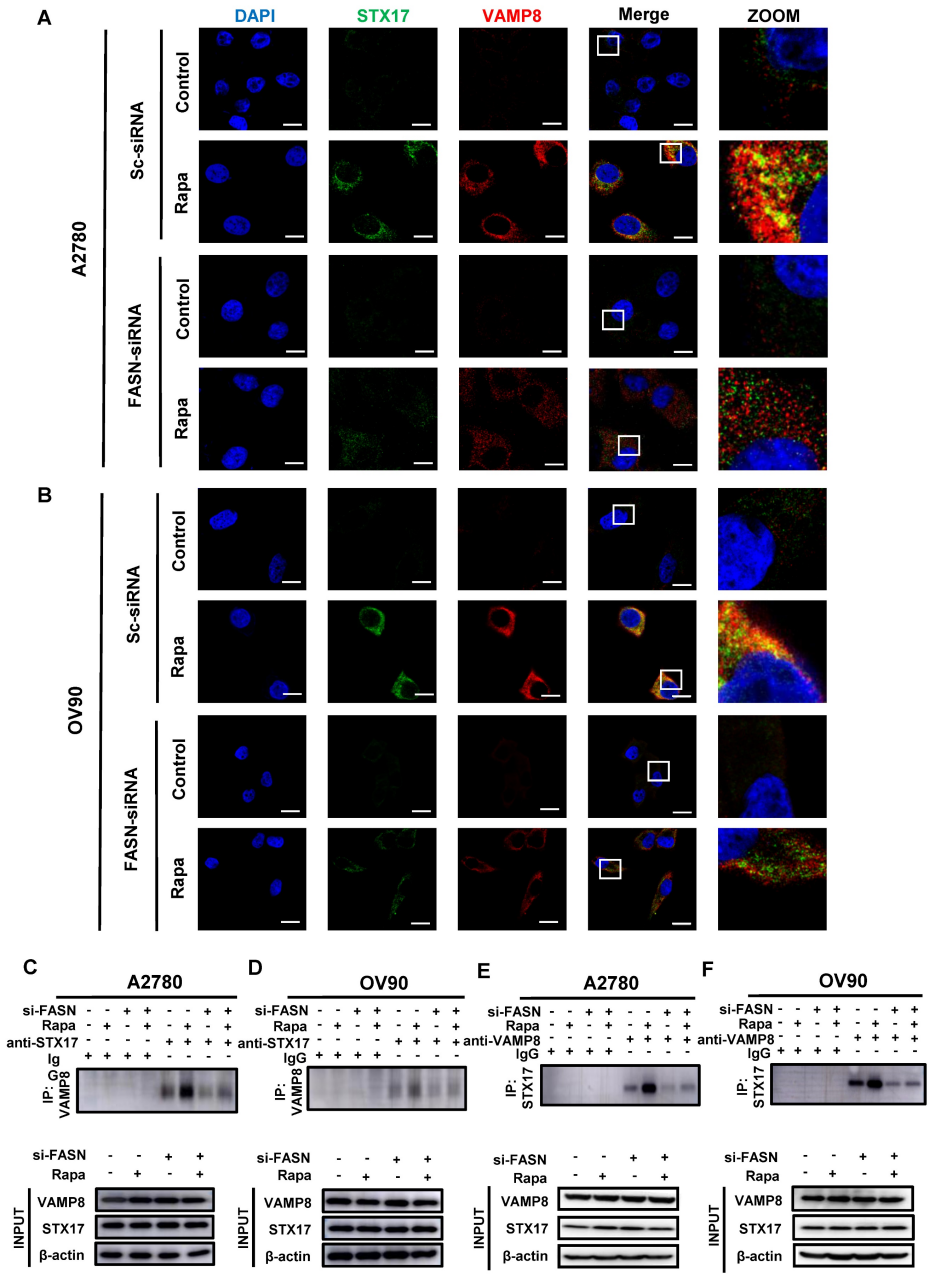
** FASN silencing suppresses Rapa-induced SNARE complexes formation.** (A, B) Representative images showing STX17 (green) and VAMP8 (red) in A2780 and OV90 cells in the presence or absence of rapamycin (1 μM) treatment for 6 h under normal or silenced FASN conditions. Scale bar: 10 μm. (C, D) A2780 and OV90 cell lysates were prepared by strong RIPA lysis and co-immunoprecipitated by STX17 antibody with IgG as negative control. (E, F) Co-immunoprecipitation assay using VAMP8 as bait protein demonstrated the interaction between VAMP8 and STX17. n = 3 in the staining experiments, and representative pictures are shown.

We previously observed that MK8722 could affect the FASN expression levels and noticed a failure in the formation of autophagic lysosomes in some experiments. Therefore, we hypothesized that FASN may influence the formation of the STX17-SNAP29-VAMP8 complex and act as a pivotal factor in OC progression. To briefly examine this hypothesis, data of OC patients were downloaded from the TCGA-OA database, the correlation between FASN and STX17 and VAMP8 was analyzed using Pearson correlation analysis (Figure S4E), a strong correlation was found between them (STX17: R=0.43, P=6.2e-21; VAMP8: R=0.-15, P=0.0026). Subsequently, the knockdown efficiency was verified by qRT-PCR analysis using transfection of siRNA-1, -2, and -3 to steadily silence FASN at the transcriptional level. The knockdown efficiency of siRNA-1, -2, and -3 was not significantly different in A2780 cells, but siRNA-2 was slightly more efficient than siRNA-1 and siRNA-3 in OV90 cells (Figure S4F). Therefore, siRNA-2 was selected for subsequent experiments to silence FASN in the two EOC cell lines. The co-localization of STX17 and VAMP8 was assessed under FASN silencing or non-silencing conditions with or without rapamycin treatment by immunofluorescence (Figure 5A, B). As anticipated, extensive yellow areas indicating co-localization between STX17 and VAMP8 were observed in the FASN-normal group treated with rapamycin, while few yellow areas were detected in the FASN-knockout group with rapamycin, significantly reducing the formation of the STX17-SNAP29-VAMP8 complex (Figure S4G). The interaction between STX17 and VAMP8 was further validated using the immunoprecipitation (Co-ip) method. Endogenous STX17 was immunoprecipitated from A2780 cell lysates using a VAMP8 antibody. The immunoprecipitation of VAMP8 with the STX17 antibody provided further confirmation of this interaction (Figure 5C, E). This result was also confirmed in OV90 cells (Figure 5D, F). Additionally, a proximity ligation assay (PLA) was conducted to further validate this interaction and shown in Figure S4H-J, strong red PLA signals were observed in the FASN wild-type group treated with Rapamycin, while they were scarce in the FASN knockout group treated with Rapamycin. These results collectively suggest a correlation between FASN and the STX17-SNAP29-VAMP8 complex.

### MK8722 represses the growth of the mouse EOC xenograft model

**Figure 6:**
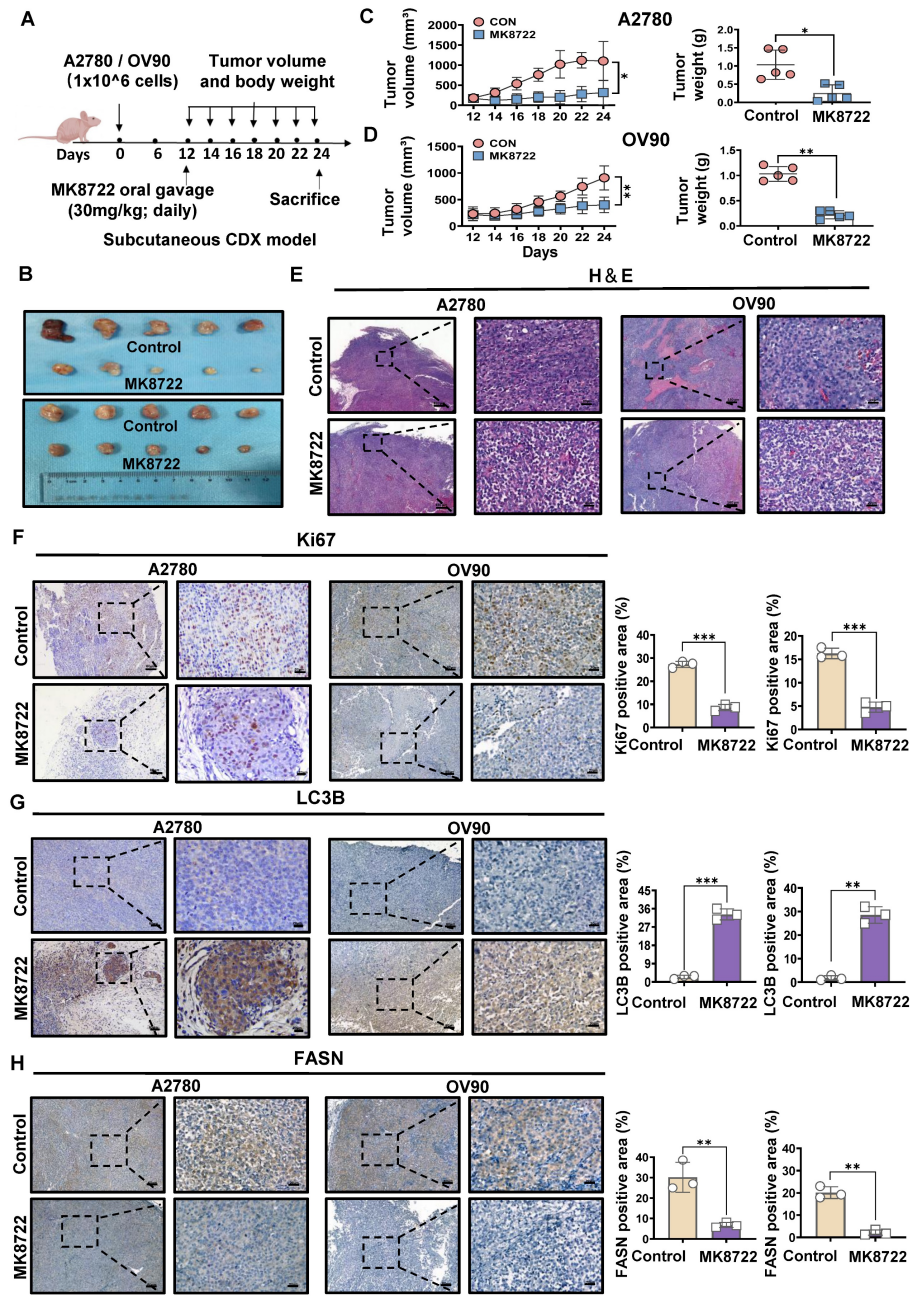
** MK8722 represses the growth of the mouse EOC xenograft model.** (A) BALB/c nude mice were subcutaneously injected with A2780 or OV90 cells. MK or a mixture of dimethyl sulfoxide and castor oil was administered daily by gavage starting from day 12. (B) Tumor volumes in the MK treatment group compared to the control group are shown. (C, D) Subcutaneous tumor size was measured every 2 days in nude mice. Volume = (length×width^2^)/2. The weight of excised tumors was measured. (E) H&E staining of paraffin sections from tumors. Scale bar: 200 μm. (F-H) IHC staining for Ki67, LC3B and FASN. Quantitative analysis of positive staining areas (brown). Scale bars: 20 μm, 80 μm. Results are presented as mean ± SD; * *p* < 0.05, ** *p* < 0.01, *** *p* < 0.001. The results represent the mean values from three independent experiments (n = 3), and representative pictures are shown.

To further investigate whether MK8722 could inhibit the outgrowth of EOC cells *in vitro* by disrupting autophagic flux, we established a mouse EOC subcutaneous transplantation tumor model. The therapeutic effects of MK8722 on EOC were evaluated *in vivo*. Nude mice were randomly divided into four groups and subcutaneously injected with A2780 and OV90 cells in two groups, respectively. The experimental group received MK8722 (30 mg/kg, orally) starting from day 12 after injection, while the control group received a mixture of dimethyl sulfoxide and castor oil every two days for 12 days (Figure 6A). The mean tumor volume and weight were significantly reduced in the MK8722-treated group compared to the control group (Figure 6B-D). However, there was no significant difference in weight between the groups (Figure S5A, B). H&E staining and IHC (Ki67) were performed on tumor specimens from the experimental animals to assess the morphological changes induced by MK8722 and its effect on tumor growth *in vivo*. H&E staining of tumor tissue sections from the mice treated with MK8722 showed pronounced morphological alterations with signs of necrosis (Figure 6E). Ki67 (brown) was clearly lower in the MK8722 therapy group than in the comparison group (Figure 6F). To verify whether autophagic vesicle-lysosome fusion was inhibited *in vivo*, tumor tissue sections from mice were stained with IHC (LC3B) and oil red O staining. The results of IHC showed extensive staining of LC3B in the experimental group (Figure 6G). Oil red O staining revealed a significant increase in the orange-red area within the tissue, indicating lipid droplet accumulation upon treatment with MK8722 (Figure S5C). However, the staining area of the FASN in the experimental group significantly decreased (Figure 6H). Blood specimens in both groups were obtained, and plasma was tested for blood biochemical parameters (Total Protein, GLU, and ALT) (Figure S5D, E). In addition, samples of the heart, liver, spleen, lung, and kidneys of the two groups of nude mice were stained with H&E (Figure S5F). The results showed that MK8722 had no significant impacts or alternative side effects on the functions of vital organs, such as the heart, liver, spleen, lung, and kidneys in mice. The findings from the *in vivo* experiments were consistent with those from the *in vitro* experiments.

## Discussion

Novel and potent autophagic inhibitors have been extensively investigated due to their potential role in anti-cancer treatment regimes, as they might be able to eliminate chemotherapeutic resistance. MK8722 is a novel suppressor of autophagy/mitochondrial autophagy, reported for the first time in this study. Our results indicated that MK8722 could activate early-stage autophagy while inhibiting late-stage autophagy through FASN-dependent reprogramming of lipid metabolism, leading to the death of human EOC cells. More importantly, we showed that modulation of FASN had profound impacts on the outcomes of SNAREs, suggesting that FASN might provide a molecular pathway from lipid metabolism to autophagy, and thus be a promising therapeutic target for EOC.

In this study, we utilized network pharmacology and bioinformatics analysis to predict the pathway enrichment of MK8722 in EOC. Our analysis revealed associations with pathways such as glioma, carbon metabolism, phagosome, ROS, and lipid metabolism. Given that several studies have highlighted the "lipid addiction" phenotype of ovarian cancer 41-43, we focused on the lipid metabolism pathway. Tumor cells undergo metabolic disturbances characterized by rapid growth and biosynthesis, leading to the accumulation of metabolic intermediates 44. Elevated glutamine metabolism is a common metabolic shift in cancer 45-47. Glycerol or sterol skeletons give rise to triglycerides (TG) or sterol esters (SE), which are subsequently deposited in lipid droplets. Hence, disruption of controlled lipid supply processes could hinder cell survival. While there has been significant attention on the targeting of cholesterol and its metabolites in tumor development, altered fatty acid (FA) metabolism in cancer cells has received less focus but is gradually gaining recognition. Among the enzymes involved in FA metabolism, FASN plays a crucial role in de novo FA synthesis. FASN is a multifunctional homodimeric protein with six different enzymatic activities and structural domains 48-50. Overexpression of FASN has been reported in solid tumors and hematopoietic tumors, including non-small cell lung cancer, breast cancer, ovarian cancer, prostate cancer, and lymphoma 51, which is consistent with the results of our on-target analysis. In subsequent validation experiments, we observed a significant reduction in FASN expression following MK8722 treatment, along with the accumulation of lipid droplets and triglycerides with opposing effects. The formation, fusion, enlargement, or shrinkage of lipid droplets can be highly regulated by the organism to meet overall metabolic demands. Here, we propose that MK8722 partially inhibits FASN, resulting it still functioning at a slower pace. Inhibition of pathways involved in lipid droplet consumption leads to their accumulation. Two pathways are known for triglyceride metabolism within lipid droplets, cytoplasmic lipolysis (lipolysis), and lysosome-mediated autophagy (lipophagy), the latter which involves the hydrolysis of lipid droplets by lysosomal acidic lipase following engulfment by autophagic vesicles 32, 52. It is essential to clarify which lipid droplet consumption process is suppressed. In our study, TEM revealed an intriguing phenomenon involving numerous autophagic vesicles, fused mitochondria with autophagosomes, and a limited number of autophagic lysosomes, suggesting the possibility of lipophagy occurring. The accumulation of lipid droplets indicates that lipophagy may be blocked, thereby preventing autophagic flux, a measure of autophagic activity. As autophagy is an important process through which cells maintain cellular homeostasis by degrading and clearing damaged or excessive cellular components, impaired autophagic flux can lead to the accumulation of defective organelles and cellular elements, ultimately leading to cell death 53. Multiple studies have demonstrated that various stages of autophagy can be inhibited by drugs, some which work in the early stages of autophagy, such as LY294002 and 3-MA injure class III phosphatidylinositol 3-kinase (PIK3C3), by halting the initiation of autophagy 54, while others such as bafilomycin A1 and chloroquine act on late-stage autophagy, via either inhibiting autophagic lysosome production or silencing the ability to lyse the autophagosome 55. Currently, the most appropriate drugs to suppress autophagy by lysosomal alkalization are antimalarial drugs (CQ and HCQ) 44. Our study revealed that MK8722 could inhibit late-stage autophagy by disrupting autophagosome-lysosome fusion, which is a prerequisite for heathy autophagic flux, leading to the accumulation of autophagosomes/mitophagosomes and lipid droplets.

To summarize, MK8722 could arrest lipophagy, resulting in the synthesis of lipid droplets; inhibit mitophagy, leading to a significant accumulation of reactive oxygen species (ROS) which affected mitochondrial mitosis and significantly accumulate autophagic substrates. To further validate these findings, we conducted co-localization experiments of lipid droplets and autophagosomes, as well as measurements of mitochondrial ROS and mitochondrial membrane potential.

Although MK8722 could act similarly to CQ and HCQ in late-stage autophagy, the mechanism by which MK8722 inhibits autophagosome-lysosome fusion is remarkably different from the latter. As described previously, CO and HCQ achieve their objectives by disrupting lysosomal acidification. Building on the results of the present study, we discovered a secondary pathway through which MK8722 exerts its therapeutic effect. Autophagosome-lysosome fusion is governed by multiple factors, including genes and signaling pathways. Recently, the assembly of membrane bridge complexes formed by soluble N-ethylmaleimide-sensitive factor attachment protein receptors (SNAREs) has been identified as drivers of autophagosome-lysosome fusion. SNARE complexes have been reported to draw membranes together in a zipper-like fashion, using autophagosome-localized Q-SNAREs, such as STX17 and SNAP29, and lysosome-localized R-SNAREs, such as VAMP8 or VAMP7. During autophagy, syntaxin 17 (STX17) is recruited to the autophagosome as a membrane-spanning SNARE protein, and it binds to SNAP29 through a close-packed hairpin-type structure to form a junior complex. Then, SNAP29 adheres to vesicle-associated membrane protein 8 (VAMP8) through covalent bonds, mediating the membrane fusion process 38, 39, 56. This indicates that lysosomes not only act as degradation stations during autophagy but also deliver SNARE proteins involved in membrane fusion 57. Recent research has also highlighted the involvement of YKT6 and ATG14 in autophagosome-lysosome fusion. ATG14, located at the early stages of autophagy, enhances autophagosome-lysosome fusion by increasing the stability of the STX17-SNAP29-VAMP8 complex through its interaction with the central domains of SNAREs using a coiled-coil structural domain 58. Importantly, in the present study, we found that the presence of FASN could directly contribute to the formation of potentially promoting SNARE complexes by interacting with the STX17-SNAP29-VAMP8 complex, as described in previous studies. This finding is supported by the following important observations: the correlation between FASN and STX17 and VAMP8 in OC patients was explored using data collected from the TCGA-OA database via Pearson correlation analysis, and a strong correlation was found. In our study, FASN was silenced using siRNA, autophagy was activated via rapamycin, which was measured using immunofluorescence, and the co-localization of STX17 with VAMP8 was visualized by CLSM. These results were in accordance with our hypothesis.

We performed immunoprecipitation (Co-IP) and proximity ligation assay (PLA) to further confirm our findings and found that the decrease in FASN results in a weakened composition of the SNARE complexes, thereby hindering the autophagosome-lysosom fusion. However, the mechanism underlying the reaction of FASN with the SNARE complexes should be accurately elucidated. It has been reported that acetylation occurs on key proteins participating in autophagic cargo assembly and autophagosome-lysosome fusion, such as SQSTM1/p62 and STX17. Additionally, acetylation controls autophagy at the transcriptional level by targeting histones and the transcription factor TFEB 59, 60. Indeed, this could be a potential area for our future research. It is worth emphasizing that although our results indicate that MK8722 primarily exerts its effects through negative regulation of FASN, clinical data suggest the existence of potential FASN-independent mechanisms. Further research is required to explore other factors related to the mechanism of action of MK8722, to enhance our understanding of MK8722's role in treating EOC, and to provide stronger scientific evidence for its clinical implementation.

As with all potential drugs, there remains a need to validate MK8722's efficacy in diverse populations, considering genetic and ethnic variations among real patients; while we've seen its inhibitory effects on tested cell lines, further research using ex vivo and patient-derived xenograft (PDX) will shed light on potential variations in response based on genetics and ethnicity. Safety assessments are crucial, as the clinically effective concentration of MK8722 may be different from that in cell and animal models. Future research should comprehensively evaluate its tolerability, including determining optimal concentrations for various cell lines and animal models and assessing potential off-target effects, as well as explore the synergistic, resistance-modulating therapeutic potential of MK8722 together with platinum-based chemotherapy drugs.

## Conclusions

**Figure 7:**
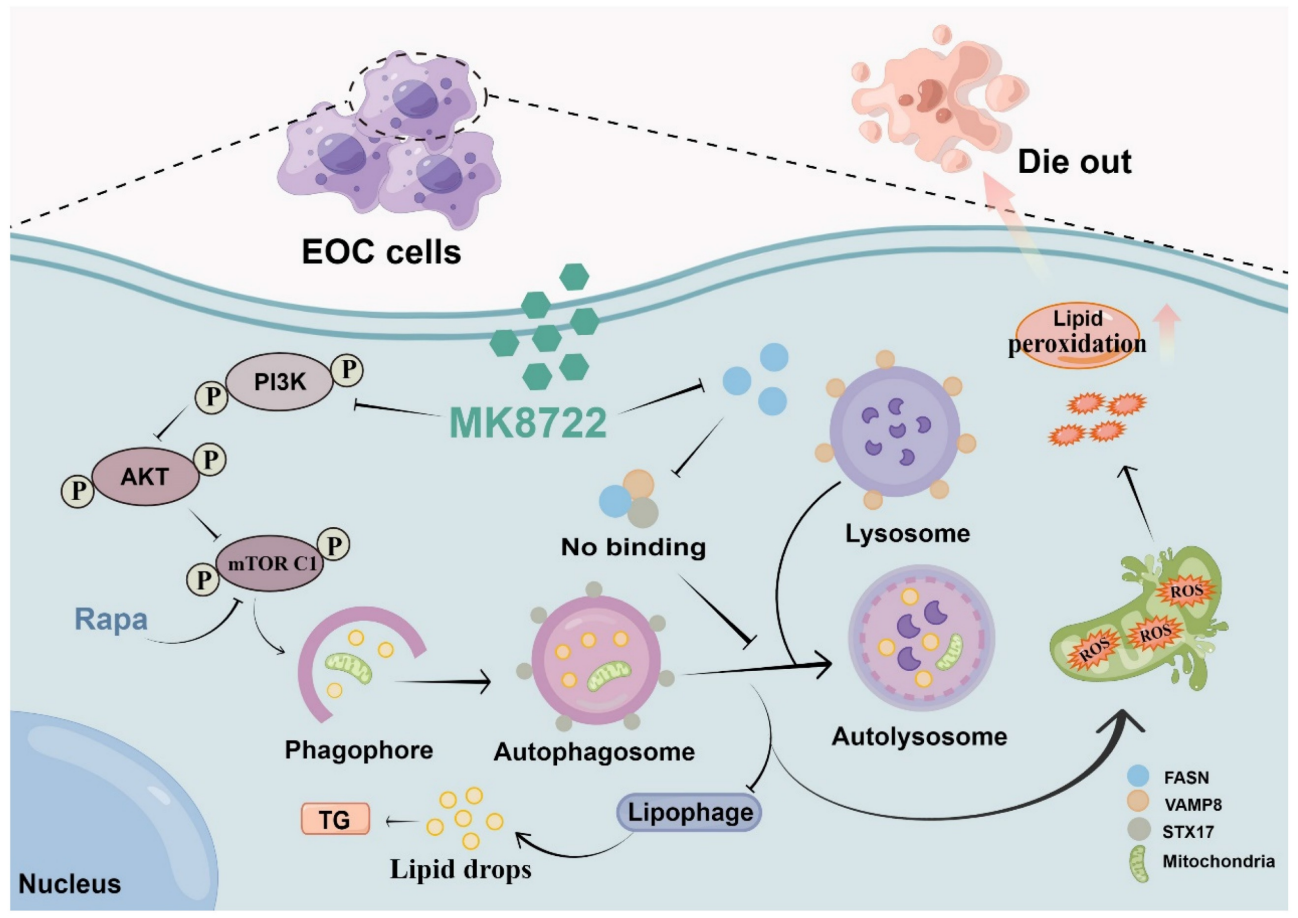
Description of the mechanism by which MK8722 activates autophagy upstream but through FASN-dependent reprogramming of lipid metabolism inhibits autophagy downstream. Created using Figdraw.

This study has, for the first time, elucidated the effects of MK8722 in attenuating the malignant biological behaviors of human EOC cells and promoting tumor cell death. Its specific mechanism involves potent inhibition of autophagosome-lysosome fusion, resulting in the accumulation of autophagosomes/lipid droplets/ROS. Importantly, it was found that MK8722 inhibits autophagy by attenuating the interaction between FASN and the SNARE complexes (Figure 7). Currently, we have conducted in-depth and comprehensive investigations at the cellular level, which provide strong support for subsequent tissue-level studies. Consequently, we strongly believe that MK8722 has excellent clinical applications after further optimization and assessment.

## Supplementary Material

Supplementary figures.

## Abbreviations

ATG: recombinant autophagy related protein
AF: Alexa Fluor
AKT: sinks serine/threonine kinase
AMPK: AMP-activated protein kinase
BSA: bovine serum albumin
BECN1: *recombinant beclin 1*
Co., Ltd.: *company limited*
CCK-8: cell counting kit-8
CQ: chloroquine
CLSM: confocal laser scanning microscopy
CO-ip: Co-immunoprecipitation
DCFH-DA: 2,7-dichlorofluorescein diacetate
DAPI: 4′,6-diamidino-2-phenylindole
DAB: 3,3'-diaminobenzine
2D: two-dimensional
3D: three-dimensional
EOC: epithelial ovarian cancer
EGFP: enhanced green fluorescent protein
FASN: fatty acid synthase
GAPDH: glyceraldehyde 3-phosphate dehydrogenase
H&E: haematoxylin-eosin
HCQ: hydroxychloroquine
IHC: immunohistochemistry
LC3: microtubule-associated protein 1 light chain 3
LAMP2: recombinant lysosomal associated membrane protein 2
mTOR: mammalian target of rapamycin
OCT: opti-mum cutting temperature compound
OC: ovarian cancer
PIK3C3: aphosphatidylinositol 3-kinase
PVDF: polyvinylidene difluoride
PI3K: phosphatidylinositol3-kinase
p-mTOR: phosphorylated mammalian target of rapamycin
p-AKT: phosphorylated sinks serine/threonine kinase
p-PI3K: phosphorylated phosphatidylinositol3-kinase
PCR: polymerase chain reaction
PBS: phosphate buffered saline
PE: PtdEth
PLA: proximity ligation assay
PDX: patient-derived tumor xenograft
RTCA: *real time casualty assessment*
ROS: reactive oxygen species
RFP: *red fluorescent protein*
RNA: ribonucleic acid
SPF: specific pathogen-free
SQSTM1: *sequestosome-1*
STX17: *syntaxin 17*
SEs: sterol esters
SDS-PAGE: sodium dodecyl sulfate polyacrylamide gel electrophoresis
SNARE: N-ethylmaleimide-sensitive factor attachment protein receptor
TG: triglycerides
TEM: transmission electron microscopy
TCGA: *the cancer genome atlas*
VAMP8: endobrevin(G-12)
WB: Western blotting
YKT6: YKT6 v-SNARE homolog (S. cerevisiae)
μM: μmol/L

## References

[1] Li X, Wang X (2017). The emerging roles and therapeutic potential of exosomes in epithelial ovarian cancer. Mol Cancer.

[2] Kuroki L, Guntupalli SR (2020). Treatment of epithelial ovarian cancer. BMJ.

[3] Lheureux S, Gourley C, Vergote I, Oza AM (2019). Epithelial ovarian cancer. Lancet.

[4] Armstrong DK, Alvarez RD, Bakkum-Gamez JN, Barroilhet L, Behbakht K, Berchuck A (2021). Ovarian Cancer, Version 2.2020, NCCN Clinical Practice Guidelines in Oncology. J Natl Compr Canc Netw.

[5] Buechel M, Herzog TJ, Westin SN, Coleman RL, Monk BJ, Moore KN (2019). Treatment of patients with recurrent epithelial ovarian cancer for whom platinum is still an option. Ann Oncol.

[6] Zhang J, Chen Y, Chen X, Zhang W, Zhao L, Weng L (2021). Deubiquitinase USP35 restrains STING-mediated interferon signaling in ovarian cancer. Cell Death Differ.

[7] Krzystyniak J, Ceppi L, Dizon DS, Birrer MJ (2016). Epithelial ovarian cancer: the molecular genetics of epithelial ovarian cancer. Ann Oncol.

[8] Kim KH, Lee MS (2014). Autophagy-a key player in cellular and body metabolism. Nat Rev Endocrinol.

[9] Dikic I, Elazar Z (2018). Mechanism and medical implications of mammalian autophagy. Nat Rev Mol Cell Biol.

[10] Levine B, Kroemer G (2019). Biological Functions of Autophagy Genes: A Disease Perspective. Cell.

[11] Mizushima N, Komatsu M (2011). Autophagy: renovation of cells and tissues. Cell.

[12] Levy JMM, Towers CG, Thorburn A (2017). Targeting autophagy in cancer. Nat Rev Cancer.

[13] Mizushima N, Levine B (2020). Autophagy in Human Diseases. N Engl J Med.

[14] Ishaq M, Ojha R, Sharma AP, Singh SK (2020). Autophagy in cancer: Recent advances and future directions. Semin Cancer Biol.

[15] Ferreira PMP, Sousa RWR, Ferreira JRO, Militao GCG, Bezerra DP (2021). Chloroquine and hydroxychloroquine in antitumor therapies based on autophagy-related mechanisms. Pharmacol Res.

[16] Emdad L, Bhoopathi P, Talukdar S, Pradhan AK, Sarkar D, Wang XY (2020). Recent insights into apoptosis and toxic autophagy: The roles of MDA-7/IL-24, a multidimensional anti-cancer therapeutic. Semin Cancer Biol.

[17] Jiang J, Zhang L, Chen H, Lei Y, Zhang T, Wang Y (2020). Regorafenib induces lethal autophagy arrest by stabilizing PSAT1 in glioblastoma. Autophagy.

[18] Patra S, Mishra SR, Behera BP, Mahapatra KK, Panigrahi DP, Bhol CS (2022). Autophagy-modulating phytochemicals in cancer therapeutics: Current evidences and future perspectives. Semin Cancer Biol.

[19] Myers RW, Guan HP, Ehrhart J, Petrov A, Prahalada S, Tozzo E (2017). Systemic pan-AMPK activator MK-8722 improves glucose homeostasis but induces cardiac hypertrophy. Science.

[20] Pang ZD, Wang Y, Song Z, She G, Ma XZ, Sun X (2021). AMPK upregulates K(Ca)2.3 channels and ameliorates endothelial dysfunction in diet-induced obese mice. Biochem Pharmacol.

[21] Zhou X, Muise ES, Haimbach R, Sebhat IK, Zhu Y, Liu F (2019). PAN-AMPK Activation Improves Renal Function in a Rat Model of Progressive Diabetic Nephropathy. J Pharmacol Exp Ther.

[22] Svensson RU, Parker SJ, Eichner LJ, Kolar MJ, Wallace M, Brun SN (2016). Inhibition of acetyl-CoA carboxylase suppresses fatty acid synthesis and tumor growth of non-small-cell lung cancer in preclinical models. Nat Med.

[23] Pineda CT, Ramanathan S, Fon Tacer K, Weon JL, Potts MB, Ou YH (2015). Degradation of AMPK by a cancer-specific ubiquitin ligase. Cell.

[24] Faubert B, Boily G, Izreig S, Griss T, Samborska B, Dong Z (2013). AMPK is a negative regulator of the Warburg effect and suppresses tumor growth in vivo. Cell Metab.

[25] Hu Y, Chen H, Zhang L, Lin X, Li X, Zhuang H (2021). The AMPK-MFN2 axis regulates MAM dynamics and autophagy induced by energy stresses. Autophagy.

[26] Jia J, Abudu YP, Claude-Taupin A, Gu Y, Kumar S, Choi SW (2019). Galectins control MTOR and AMPK in response to lysosomal damage to induce autophagy. Autophagy.

[27] Garcia D, Shaw RJ (2017). AMPK: Mechanisms of Cellular Energy Sensing and Restoration of Metabolic Balance. Mol Cell.

[28] Lee JH, Mohan CD, Deivasigamani A, Jung YY, Rangappa S, Basappa S (2020). Brusatol suppresses STAT3-driven metastasis by downregulating epithelial-mesenchymal transition in hepatocellular carcinoma. J Adv Res.

[29] Guo S, Zhang J, Wei C, Lu Z, Cai R, Pan D (2020). Anticancer effects of brusatol in nasopharyngeal carcinoma through suppression of the Akt/mTOR signaling pathway. Cancer Chemother Pharmacol.

[30] Walther TC, Chung J, Farese RV Jr (2017). Lipid Droplet Biogenesis. Annu Rev Cell Dev Biol.

[31] Thiam AR, Ikonen E (2021). Lipid Droplet Nucleation. Trends Cell Biol.

[32] Zechner R, Madeo F, Kratky D (2017). Cytosolic lipolysis and lipophagy: two sides of the same coin. Nat Rev Mol Cell Biol.

[33] Dou Z, Xu C, Donahue G, Shimi T, Pan JA, Zhu J (2015). Autophagy mediates degradation of nuclear lamina. Nature.

[34] Romao S, Munz C (2014). LC3-associated phagocytosis. Autophagy.

[35] Kabeya Y, Mizushima N, Ueno T, Yamamoto A, Kirisako T, Noda T (2000). LC3, a mammalian homologue of yeast Apg8p, is localized in autophagosome membranes after processing. EMBO J.

[36] Mizushima N, Yoshimori T (2007). How to interpret LC3 immunoblotting. Autophagy.

[37] Teresak P, Lapao A, Subic N, Boya P, Elazar Z, Simonsen A (2022). Regulation of PRKN-independent mitophagy. Autophagy.

[38] Shen Q, Shi Y, Liu J, Su H, Huang J, Zhang Y (2021). Acetylation of STX17 (syntaxin 17) controls autophagosome maturation. Autophagy.

[39] Huang H, Ouyang Q, Zhu M, Yu H, Mei K, Liu R (2021). mTOR-mediated phosphorylation of VAMP8 and SCFD1 regulates autophagosome maturation. Nat Commun.

[40] Tian X, Teng J, Chen J (2021). New insights regarding SNARE proteins in autophagosome-lysosome fusion. Autophagy.

[41] Yang CS, Matsuura K, Huang NJ, Robeson AC, Huang B, Zhang L (2015). Fatty acid synthase inhibition engages a novel caspase-2 regulatory mechanism to induce ovarian cancer cell death. Oncogene.

[42] Jiang L, Wang H, Li J, Fang X, Pan H, Yuan X (2014). Up-regulated FASN expression promotes transcoelomic metastasis of ovarian cancer cell through epithelial-mesenchymal transition. Int J Mol Sci.

[43] Nieman KM, Kenny HA, Penicka CV, Ladanyi A, Buell-Gutbrod R, Zillhardt MR (2011). Adipocytes promote ovarian cancer metastasis and provide energy for rapid tumor growth. Nat Med.

[44] Kreuzaler P, Watson CJ (2012). Killing a cancer: what are the alternatives?. Nat Rev Cancer.

[45] Huang M, Xiong D, Pan J, Zhang Q, Sei S, Shoemaker RH (2022). Targeting Glutamine Metabolism to Enhance Immunoprevention of EGFR-Driven Lung Cancer. Adv Sci (Weinh).

[46] Edwards DN, Ngwa VM, Raybuck AL, Wang S, Hwang Y, Kim LC (2021). Selective glutamine metabolism inhibition in tumor cells improves antitumor T lymphocyte activity in triple-negative breast cancer. J Clin Invest.

[47] van Geldermalsen M, Wang Q, Nagarajah R, Marshall AD, Thoeng A, Gao D (2016). ASCT2/SLC1A5 controls glutamine uptake and tumour growth in triple-negative basal-like breast cancer. Oncogene.

[48] Li Z, Zhang H (2016). Reprogramming of glucose, fatty acid and amino acid metabolism for cancer progression. Cell Mol Life Sci.

[49] Ma Y, Temkin SM, Hawkridge AM, Guo C, Wang W, Wang XY (2018). Fatty acid oxidation: An emerging facet of metabolic transformation in cancer. Cancer Lett.

[50] Koundouros N, Poulogiannis G (2020). Reprogramming of fatty acid metabolism in cancer. Br J Cancer.

[51] Falchook G, Infante J, Arkenau HT, Patel MR, Dean E, Borazanci E (2021). First-in-human study of the safety, pharmacokinetics, and pharmacodynamics of first-in-class fatty acid synthase inhibitor TVB-2640 alone and with a taxane in advanced tumors. EClinicalMedicine.

[52] Schulze RJ, Sathyanarayan A, Mashek DG (2017). Breaking fat: The regulation and mechanisms of lipophagy. Biochim Biophys Acta Mol Cell Biol Lipids.

[53] Yang Y, Kong B, Jung Y, Park JB, Oh JM, Hwang J (2018). Soluble N-Ethylmaleimide-Sensitive Factor Attachment Protein Receptor-Derived Peptides for Regulation of Mast Cell Degranulation. Front Immunol.

[54] Bernard M, Yang B, Migneault F, Turgeon J, Dieude M, Olivier MA (2020). Autophagy drives fibroblast senescence through MTORC2 regulation. Autophagy.

[55] Mauthe M, Orhon I, Rocchi C, Zhou X, Luhr M, Hijlkema KJ (2018). Chloroquine inhibits autophagic flux by decreasing autophagosome-lysosome fusion. Autophagy.

[56] Bernard A, Klionsky DJ (2015). Toward an understanding of autophagosome-lysosome fusion: The unsuspected role of ATG14. Autophagy.

[57] Ballabio A, Bonifacino JS (2020). Lysosomes as dynamic regulators of cell and organismal homeostasis. Nat Rev Mol Cell Biol.

[58] Diao J, Liu R, Rong Y, Zhao M, Zhang J, Lai Y (2015). ATG14 promotes membrane tethering and fusion of autophagosomes to endolysosomes. Nature.

[59] Shvedunova M, Akhtar A (2022). Modulation of cellular processes by histone and non-histone protein acetylation. Nat Rev Mol Cell Biol.

[60] Xu Y, Wan W (2023). Acetylation in the regulation of autophagy. Autophagy.

