# Supplementary material for: MK8722 initiates early-stage autophagy while inhibiting late-stage autophagy via FASN-dependent reprogramming of lipid metabolism
Source: Theranostics. 2024 Jan 1;14(1):75–95. doi: 10.7150/thno.83051 (PMC10750195; doi:10.7150/thno.83051)
Supplement: Supplementary file 1 — Supplementary figures. [file thnov14p0075s1.pdf]

Supplementary Information

for

MK8722 initiates early-stage autophagy while inhibiting late-stage autophagy via FASN-dependent reprogramming of lipid metabolism

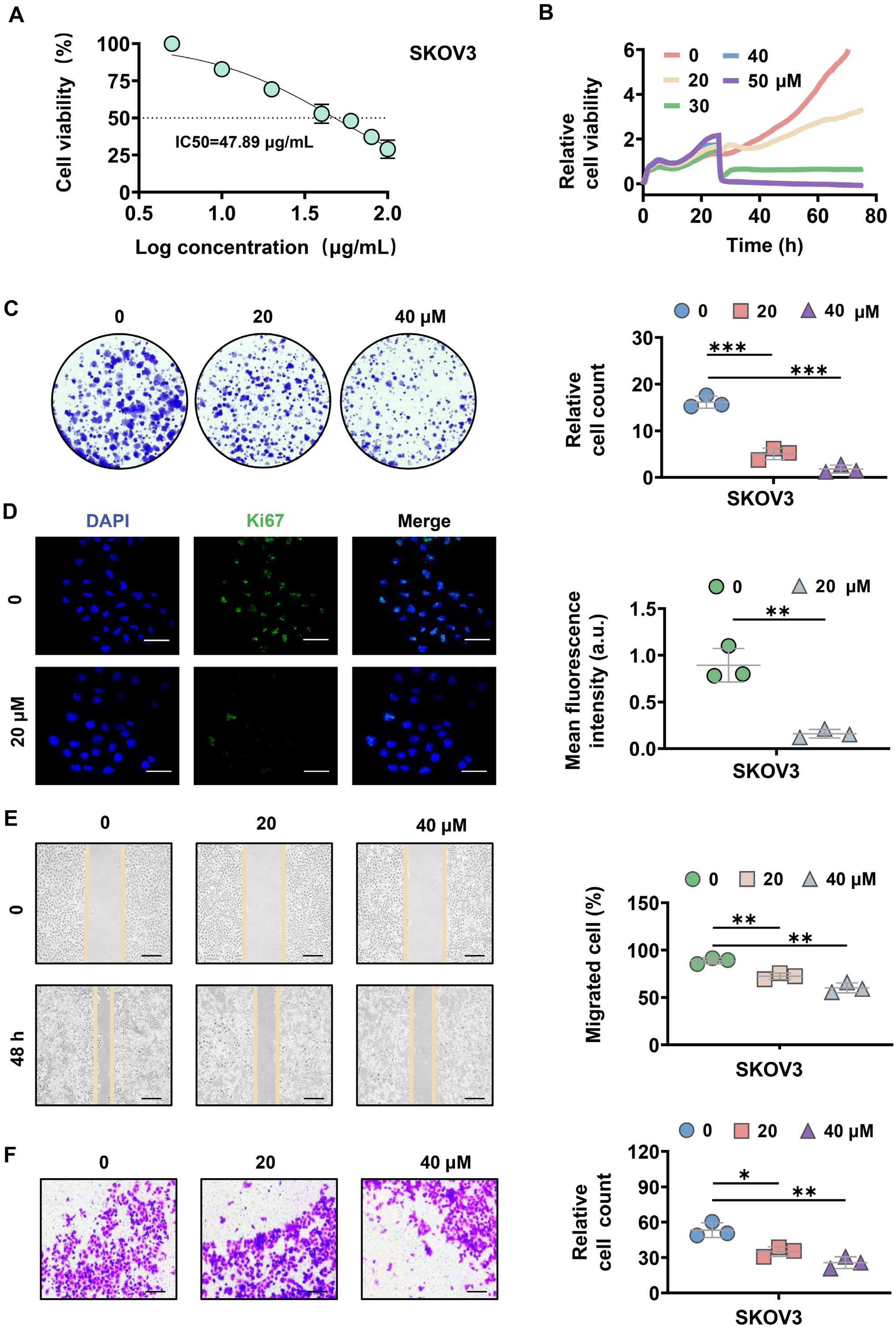

Figure S1. MK8722 inhibits the growth and migration of EOC cells *in vitro.*

(A) SKOV3 cell was incubated with 5, 10, 20, 40, 60, 80, 100 μM MK8722 (MK) or an equal volume of DMEM for 48 h, respectively. (B) SKOV3 cell was exposed to MK, and real-time cell activity was measured using RTCA. (C) SKOV3 cell was incubated with MK (20 μM, 40 μM) or an equal volume of DMEM culture solution for 48 h, and a colony formation assay was conducted. (D) SKOV3 cell was cultured with the presence of MK (20 μM) or an equal volume DMEM culture for 48 h and then subjected to Ki-67 immunofluorescence analysis. Scale bar: 15 μm. (E) SKOV3 cell was plated with 20 μM, 40 μM MK or DMEM for 48 h to observe wound healing. Scale bar: 100 μm. (F) Transwell migration assay was used to evaluate the effect of 20 μM and 40 μM MK on the migration of SKOV3 cell. Scale bar: 100 μm. Results were exhibited as mean ± SD; * $p < 0.05$, ** $p < 0.01$, *** $p < 0.001$. The results represent the mean value from three independent experiments (n = 3), and representative pictures are shown.

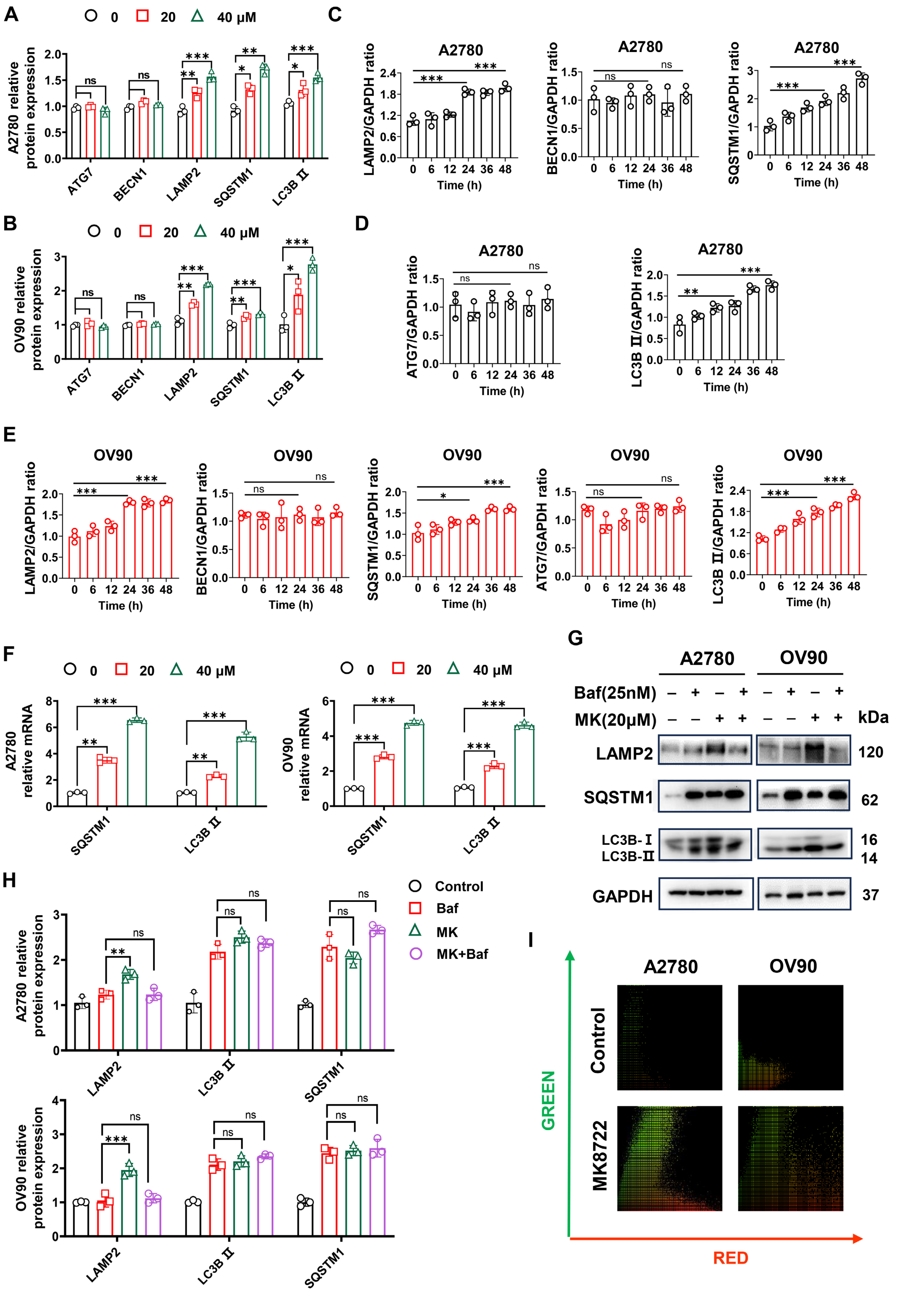

Figure S2. MK8722 enhances LC3B-II stability and spot formation in EOC cells and inhibits lipophagy.

(A, B) Western blot analysis and quantification of ATG7, BECN1, LAMP2, P62/SQSTM1, and LC3B II/I protein expression in A2780 and OV90 cells exposed to different concentrations of MK for 48 h. (C-E) Western blot analysis and quantification of protein expression in A2780 and OV90 cells exposed to different time of MK (20 mM). (F) qPCR analysis of p62/SQSTM1 and LC3B mRNA expression in A2780 and OV90 cells treated with different concentrations of MK for 48 h. (G, H) Cells were treated without or with MK (20 mM) in the presence or absence of 25 nM Baf for 24 h; the expression of SQSTM1, LC3B-II and LAMP2 was analyzed by western blot. (I) Quantitative analysis of the relationship between EGFP-LC3 and Nile red in A2780 and OV90 cells. Results were exhibited as mean ± SD; NS, not significant, * $p < 0.05$, ** $p < 0.01$, *** $p < 0.001$. The results represent the mean value from three independent experiments (n = 3), and representative pictures are shown.

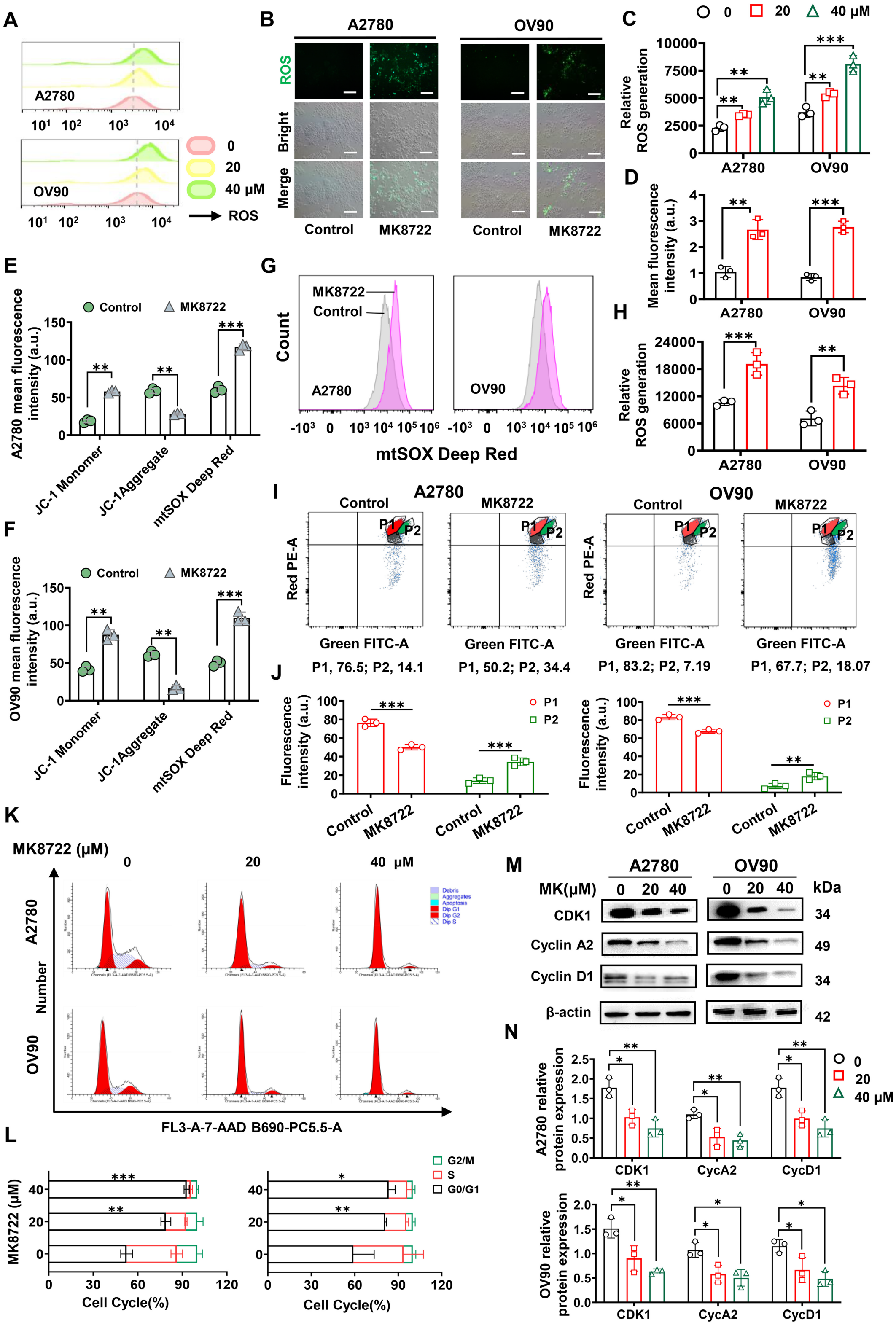

1

2 **Figure S3. MK8722 leads to ROS accumulation and affects mitosis.**

(A, C) Changes in intracellular ROS fluorescence intensity in A2780 and OV90 cells treated with or without MK (20 μM) were shown using DCFH-DA as a probe under inverted fluorescence microscopy. Scale bar: 100 μm. (B, D) Flow cytometry analysis demonstrated changes in intracellular ROS levels in both cell lines with or without MK (20, 40 μM) treatment. (E, F) Quantitative analysis of JC-1 monomer, JC-1 aggregate and mtSOX fluorescence intensity changes. (G, H) Flow cytometry analysis showed that mitochondrial ROS changes in both cell lines with or without MK (20 μM) treatment. (I, J) Flow cytometry analysis showed that JC-1 aggregate (P1), JC-1 monomer (P2) changes in both cell lines with or without MK (20 μM) treatment. (K, L) Flow cytometry analysis demonstrated changes in cell cycle in both cell lines with or without MK (20, 40 μM) treatment. (M, N) Expression of cell cycle-related proteins were determined by western blot. The protein expressions were quantitatively analyzed. Results were exhibited as mean ± SD; * $p < 0.05$, ** $p < 0.01$, *** $p < 0.001$. The results represent the mean value from three independent experiments (n = 3), and representative pictures are shown.

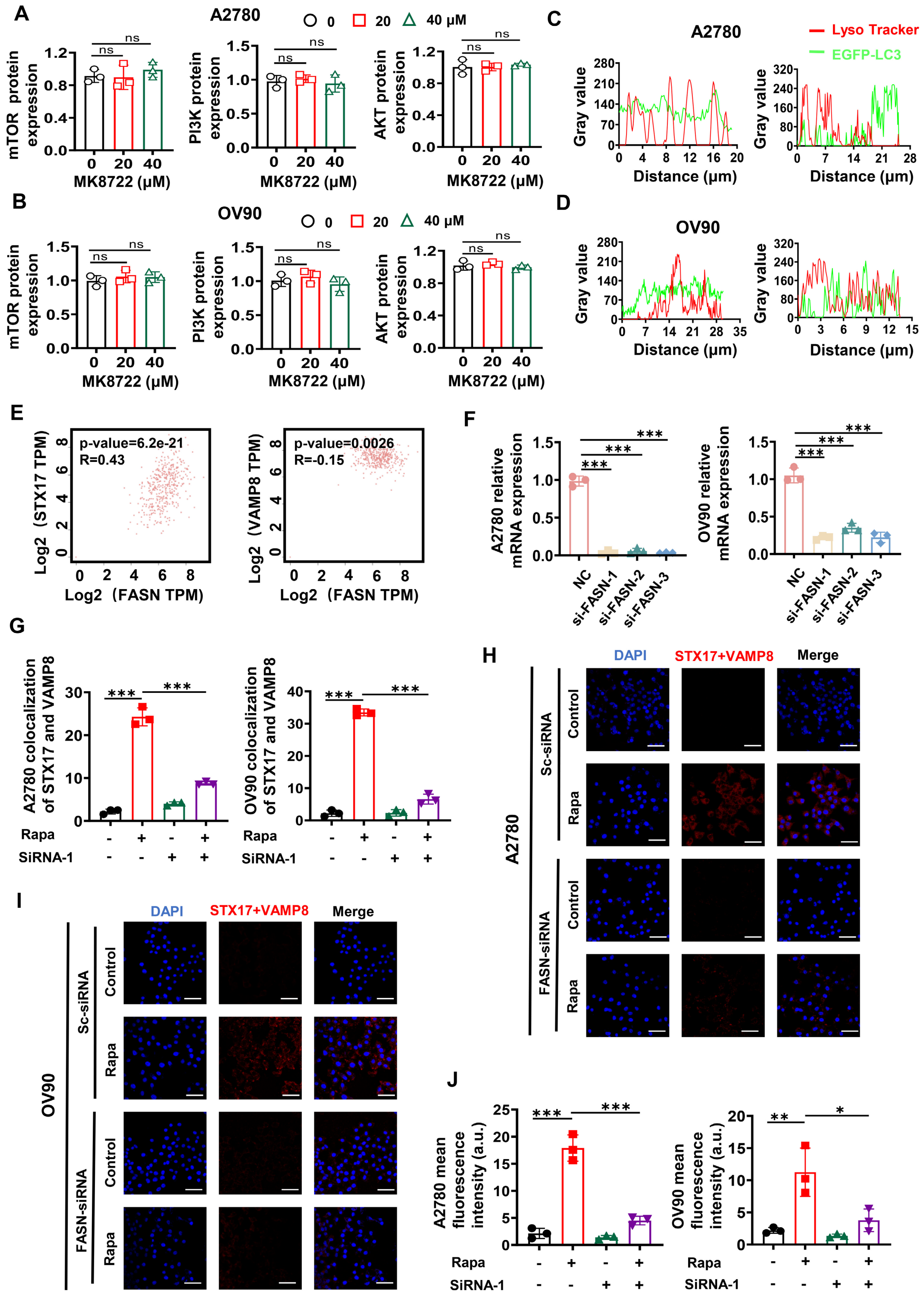

Figure S4. MK8722 promotes autophagy upstream (PI3K, AKT, and mTOR), but suppresses autophagic vesicle-lysosome fusion by FASN silencing suppressing Snare complex formation.

(A, B) Western blot analysis and quantification of mTOR, PI3K, and AKT protein expression in A2780 and OV90 cells exposed to different concentrations of MK for 48 h. (C, D) Qualitative analysis of the relationship between EGFP-LC3 and LysoTracker in A2780 and OV90 cells. Left panel shows control, right panel shows MK treatment. (E) Correlation analysis of FASN with STX17 and VAMP8 in ovarian cancer patients downloaded from the TCGA-OA database using Pearson's analysis. (F) qRT-PCR analysis of siRNA-1, 2, 3 knockdown efficiency at the transcriptional level in A2780 and OV90 cells. (G) Quantitative analysis of yellow area produced by co-localization of STX17 with VAMP8 in A2780 and OV90 , respectively. (H-J) PLA assay was performed in A2780 and OV90 cells treated with or without MK (20 μM) and quantification of red fluorescence intensity. Results were exhibited as mean ± SD; NS, not significant, * $p < 0.05$, ** $p < 0.01$, *** $p < 0.001$. The results represent the mean value from three independent experiments ($n = 3$), and representative pictures are shown.

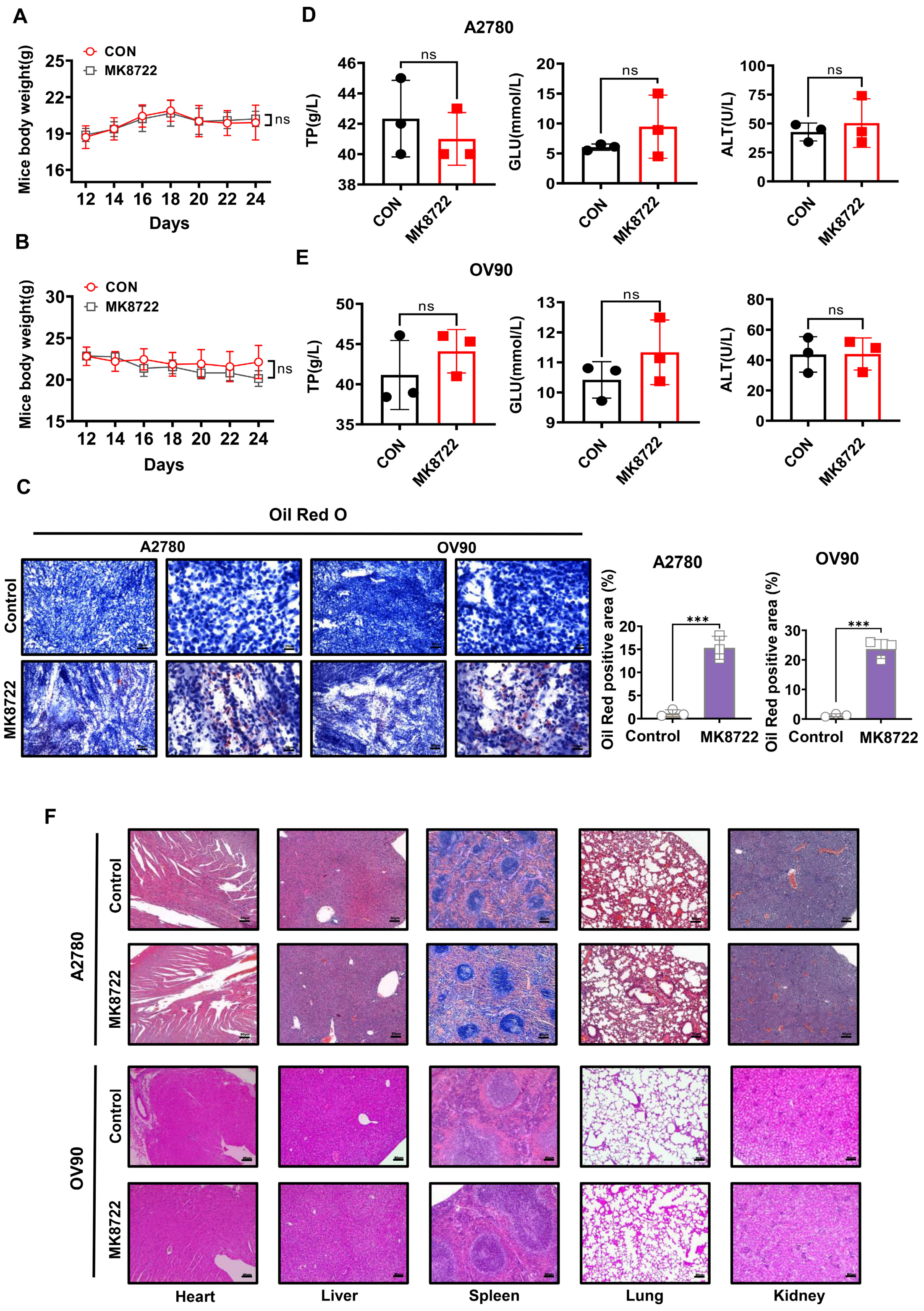

Figure S5. MK8722 causes the accumulation of lipid droplets *in vivo* and MK8722 has no adverse effects.

(A, B) Monitoring the body weight of nude mice in the control group and the experimental group. (C) Oil red O staining of frozen tumor sections. Quantitative analysis of positive staining areas (red). Scale bars: 20 μm, 80 μm. (D-E) Changes of TP, GLU and ALT in the control group and the experimental group. (F) H&E staining of paraffin sections from the heart, liver, spleen, lung, and kidney of nude mice in the experimental and control groups. Scale bar: 80 μm. Results are presented as mean ± SD; NS, not significant, *** $p < 0.001$. The results represent the mean value from three independent experiments (n = 3), and representative pictures are shown.
